# Fear in general populations: A cross-sectional study on perceived fear of common diseases, COVID-19, life events, and environmental threats in 30 countries

**DOI:** 10.7189/jogh.14.05019

**Published:** 2024-06-07

**Authors:** Jiaying Li, Vinciya Pandian, Daniel Yee Tak Fong, Kris Yuet Wan Lok, Janet Yuen Ha Wong, Mandy Man Ho, Edmond Pui Hang Choi, Patricia M Davidson, Wenjie Duan, Marie Tarrant, Jung Jae Lee, Chia-Chin Lin, Oluwadamilare Akingbade, Khalid M Alabdulwahhab, Mohammad Shakil Ahmad, Mohamed Alboraie, Meshari A Alzahrani, Anil S Bilimale, Sawitree Boonpatcharanon, Samuel Byiringiro, Muhammad Kamil Che Hasan, Luisa Clausi Schettini, Walter Corzo, Josephine M De Leon, Anjanette S De Leon, Hiba Deek, Fabio Efficace, Mayssah A El Nayal, Fathiya El-Raey, Eduardo Ensaldo-Carrasco, Pilar Escotorin, Oluwadamilola Agnes Fadodun, Israel Opeyemi Fawole, Yong-Shian Shawn Goh, Devi Irawan, Naimah Ebrahim Khan, Binu Koirala, Ashish Krishna, Cannas Kwok, Tung Thanh Le, Daniela Giambruno Leal, Miguel Ángel Lezana-Fernández, Emery Manirambona, Leandro Cruz Mantoani, Fernando Meneses-González, Iman Elmahdi Mohamed, Madeleine Mukeshimana, Chinh Thi Minh Nguyen, Huong Thi Thanh Nguyen, Khanh Thi Nguyen, Son Truong Nguyen, Mohd Said Nurumal, Aimable Nzabonimana, Nagla Abdelrahim Mohamed Ahmed Omer, Oluwabunmi Ogungbe, Angela Chiu Yin Poon, Areli Reséndiz-Rodriguez, Busayasachee Puang-Ngern, Ceryl G Sagun, Riyaz Ahmed Shaik, Nikhil Gauri Shankar, Kathrin Sommer, Edgardo Toro, Hanh Thi Hong Tran, Elvira L Urgel, Emmanuel Uwiringiyimana, Tita Vanichbuncha, Naglaa Youssef

**Affiliations:** 1 School of Nursing, Li Ka Shing Faculty of Medicine, University of Hong Kong, Hong Kong SAR, China; 2 School of Nursing, Johns Hopkins University, Baltimore, Maryland, USA; 3 School of Nursing and Health Studies, Hong Kong Metropolitan University, Hong Kong SAR, China; 4 Vice-Chancellor and Principal, University of Wollongong, Wollongong, Australia; 5 Department of Social Work, East China University of Science and Technology, Shanghai, China; 6 School of Nursing, The University of British Columbia, Kelowna British Columbia, Canada; 7 The Nethersole School of Nursing, The Chinese University of Hong Kong, Hong Kong; 8 Institute of Nursing Research, Osogbo, Osun State, Nigeria; 9 College of Medicine, Majmaah University, Al Majmaah, Saudi Arabia; 10 Department of Family & Community Medicine, College of Medicine, Majmaah University, Majmaah, Saudi Arabia; 11 Department of Internal Medicine, Al-Azhar University, Cairo, Egypt; 12 Department of Urology, College of Medicine, Majmaah University, Al Majmaah, Saudi Arabia; 13 School of Public Health, JSS Medical College, JSS AHER, Mysuru, India; 14 Department of Statistics, Chulalongkorn Business School, Bangkok, Thailand; 15 Kulliyyah of Nursing, International Islamic University, Kuantan, Malaysia; 16 Italian Association against Leukemia, Lymphoma and Myeloma, Rome Section, Italy; 17 Diálogos Guatemala, Guatemala, Guatemala; 18 School of Nursing, Centro Escolar University, Manila, Philippines; 19 Nursing Department, Faculty of Health Science, Beirut Arab University, Lebanon; 20 Italian Group for Adult Hematologic Disease, Data Center and Health Outcomes Research Unit, Rome, Italy; 21 Department of Psychology, Beirut Arab University, Lebanon; 22 Department of hepatogastroenterology and infectious diseases, Damietta faculty of medicine, Al-Azher University, Egypt; 23 Ergonomics Research Center, University of Guadalajara, Jalisco, Mexico; 24 Laboratory of Applied Prosocial Research, Department of Basic, Developmental and Educational Psychology, Autonomous University of Barcelona, Spain; 25 Faculty of Health Sciences, University of Lethbridge, Alberta, Canada; 26 Faculty of Nursing, Ladoke Akintola University of Technology, Ogbomosho, Nigeria; 27 Alice Lee Centre for Nursing Studies, National University of Singapore, Singapore; 28 School of Nursing, Wijaya Husada Health Institute, Bogor, Indonesia; 29 Department of Optometry, University of Kwazulu-Natal, Durban, South Africa; 30 Ecove, Ghaziabad, India; 31 School of Nursing, Paramedicine and Health Care Science, Charles Sturt University, New South Wales, Australia; 32 Nam Dinh University of Nursing, Nam Dinh, Vietnam; 33 Pontificia Universidad Católica de Valparaíso, School of Social Work, Valparaíso, Chile; 34 Research Department, National Commission for Medical Arbitration, Mexico, Mexico; 35 College of Medicine and Health Sciences, University of Rwanda, Kigali, Rwanda; 36 Department of Physiotherapy, Faculty of Science and Technology, São Paulo State University (UNESP), Presidente Prudente, Brazil; 37 Pharmacology and Toxicology Department, Faculty of Pharmacy, Benghazi University, Libya; 38 School of Nursing and Midwifery, College of Medicine and Health Sciences, University of Rwanda, Kigali, Rwanda; 39 Center for Language Enhancement, College of Arts and Social Sciences, University of Rwanda, Huye, Rwanda; 40 Faculty of Medicine, Alzaiem Alazhari University, Khartoum North, Sudan; 41 Faculty of Health Sciences and Sports, Macao Polytechnic University, Macao; 42 National Autonomous University of Mexico, Mexico; 43 Mental Health and Learning division, Wrexham Maelor Hospital, Wrexham, UK; 44 Medical-surgical Nursing Department, Faculty of Nursing, Cairo University, Egypt

## Abstract

**Background:**

In this study, we assessed the general population's fears towards various diseases and events, aiming to inform public health strategies that balance health-seeking behaviours.

**Methods:**

We surveyed adults from 30 countries across all World Health Organization (WHO) regions between July 2020 and August 2021. Participants rated their fear of 11 factors on an 11-point Likert scale. We stratified the data by age and gender and examined variations across countries and regions through multidimensional preference analysis.

**Results:**

Of the 16 512 adult participants, 62.7% (n = 10 351) were women. The most feared factor was the loss of family members, reported by 4232 participants (25.9%), followed by cancer (n = 2248, 13.7%) and stroke (n = 1416, 8.7%). The highest weighted fear scores were for loss of family members (mean (x̄) = 7.46, standard deviation (SD) = 3.04), cancer (x̄ = 7.00, SD = 3.09), and stroke (x̄ = 6.61, SD = 3.24). The least feared factors included animals/insects (x̄ = 3.72, SD = 2.96), loss of a mobile phone (x̄ = 4.27, SD = 2.98), and social isolation (x̄ = 4.83, SD = 3.13). Coronavirus disease 2019 (COVID-19) was the sixth most feared factor (x̄ = 6.23, SD = 2.92). Multidimensional preference analyses showed distinct fears of COVID-19 and job loss in Australia and Burundi. The other countries primarily feared loss of family members, cancer, stroke, and heart attacks; this ranking was consistent across WHO regions, economic levels, and COVID-19 severity levels.

**Conclusions:**

Fear of family loss can improve public health messaging, highlighting the need for bereavement support and the prevention of early death-causing diseases. Addressing cancer fears is crucial to encouraging the use of preventive services. Fear of non-communicable diseases remains high during health emergencies. Top fears require more resources and countries with similar concerns should collaborate internationally for effective fear management.

Fear, extending beyond just emotional response, can lead to various physical and psychological issues like cardiovascular diseases, weakened immunity, digestive problems, anxiety, depression, substance abuse, and social isolation [1–5]. It also critically drives health behaviours. For example, during the coronavirus disease 2019 (COVID-19) pandemic, fear contributed to the avoidance of health care facilities, delaying treatment for non-communicable diseases [6]. Similarly, fear associated with conditions like cancer often leads to people avoiding essential screenings [7]. This makes understanding public fears about diseases vital for shaping effective health education and patient engagement strategies which would help mitigate their negative impact on health behaviours and improve overall health outcomes.

In relation to this, comparative fear analysis offers insights into perceived disease threats, thereby informing effective risk communication by helping health communicators tailor messages to address misconceptions and provide accurate information. Additionally, recognising regional variations in fear can help improve disease surveillance, inform the design of region-specific health strategies, and generally foster global public health collaborations.

Existing research on fear of diseases like cancer and COVID-19, while diverse, lacks a consistent benchmark for cross-disease comparisons. Specifically, studies have primarily focussed on life events or non-communicable diseases, but have not adequately compared them together [4,8]. Additionally, they often neglected crucial factors such as economic status, cultural context, and health care infrastructure [9–11]. In terms of regional differences, studies on a single disease, such as a systematic review of fear of COVID-19, also showed varied results. For instance, the aforementioned review found higher fear levels in Asia compared to America, Europe, and Australia, but the reliability of these findings was undermined by a between-study heterogeneity of 98% [12]. Furthermore, researchers have hypothesised that Western countries may have greater concerns over chronic diseases than infectious ones, in contrast to many low- and middle-income countries [13]. However, this hypothesis remains untested. The lack of comprehensive cross-factors and cross-regional studies, especially within the context of the COVID-19 pandemic, highlights a significant gap in research that should be addressed through a systematic and multifactorial approach to studying fear variations across diseases and regions.

We therefore designed this study to evaluate individuals' fear levels associated with 11 common factors, with comparisons across diseases, countries, and regions through multidimensional preference analysis. Specifically, we hypothesise that there are variations in fear levels across different diseases and that the rankings of these fears differ based on regional contexts. We expect our findings could inform resource allocation and the development of support and treatments targeted at the most significant fears identified across different populations.

## METHODS

### Study design and setting

We designed this study as a cross-sectional international online survey in order to reach a diverse population across 30 countries spanning six World Health Organization (WHO) regions: Australia, Brazil, Burundi, Canada, Chile, Egypt, Guatemala, Hong Kong, India, Indonesia, Italy, Lebanon, Libya, Macau, Mainland China, Malaysia, Mexico, Nigeria, the Philippines, the Republic of Sudan, Rwanda, Saudi Arabia, Singapore, South Africa, South Korea, Spain, Thailand, UK, USA, and Vietnam. This wide geographical range ensured the comprehensive representation of different parts of the world.

### Participants and sample size

To enhance the diversity of our sample and gather a wide range of perspectives across different geographies and demographics, we combined convenience sampling with snowball sampling to recruit adults aged 18 and over from 30 countries. We determined our target sample size by estimating the prevalence of a health-related issue. Considering the most conservative scenario of a 50% prevalence rate with a 5% margin of error at a 95% confidence interval (CI), we estimated that we had to recruit approximately 385 subjects per country. Accounting for potential incomplete questionnaires, our target was to enrol at least 500 subjects in each country [14].

### Variables and measurements development

#### Socio-demographics

We retrieved the following sociodemographic variables: gender, age, country, marital status, education, employment, perceived social rank, weight, height, body mass index (BMI), pregnancy status, gestational week (if applicable), the need for regular medical follow-up before COVID-19, being a practicing health professional, having children under the age of 18 years, the number of people in the household, and house size.

#### Fear of eleven factors questionnaire

We initially developed the questionnaire for assessing 11 fear factors through an extensive literature search followed by several discussions with a multidisciplinary team of public health professionals, nurses, and nutritionists in Hong Kong. This collaborative process led to the creation of a preliminary questionnaire in English, designed to meet the study's objectives and ensure face validity. To ensure cultural acceptability, we discussed the questionnaire items with experts in specific countries and translated into the required local languages using the standard back translation process. To ensure data consistency across countries and verify that the questionnaire items were adequately understood by local groups, we piloted it with at least 10 subjects for each language version.

We instructed participants to rate their fear level on an 11-point Likert scale for various factors that if happen to them, which included common diseases, life events, and environmental threats. Specifically, common diseases included cancer, stroke, heart attack, and COVID-19. Life events included job loss, loss of family members, loss of a mobile phone, and no social life, as these factors impact an individual's life. Environmental threats included traffic accidents, crises (e.g. earthquakes, tsunamis, tornadoes, super typhoons, wars, political violence, radiation leaks, or being shot), and animal/insect (e.g. cockroaches, rats, snakes, spiders, bees, lizards, or others).

#### Validation and rigor

We also included a validation question to improve internal validity, asking participants ‘Where does the sun rise every day?’ In Nigeria, we adapted this to ‘Where is your STATE capital?’ for cultural relevance. Prior to administering language-specific questionnaires or electronic surveys, a pilot study was conducted involving at least ten participants.

#### Data collection

From 6 July 2020 to 4 August 2021, our data collection team used a multi-faceted recruitment approach across 30 participating countries. Specifically, participants were recruited through survey service providers who contacted eligible individuals from their existing databases of consenting past study participants. Additionally, the study was advertised on various social media platforms, including Facebook, WeChat, Twitter, and LinkedIn, to reach a wider audience. We also used snowball sampling techniques, particularly in universities, health clinics, and community centres, where we encouraged individuals to share the survey within their networks. A PDF-based offline electronic form was also made available for regions with limited internet access, to allow for electronic data entry into a centralised database. Respondents filled in the questionnaire anonymously. To incentivise participation, we made a donation of HKD 1 (USD 0.13) to the Red Cross in the respondent’s region for each completed questionnaire. This diverse strategy ensured an inclusive approach to data collection, incorporating both online platforms and offline electronic forms to allow access in regions with different levels of internet access.

#### Data analysis

We gathered the collected data into a master Microsoft Excel, version 16.52 (Redmond, Washington, USA) database and conducted quality control checks to detect missing responses, duplications, and discrepancies. We determined sample weights for each country based on the age and gender distribution of the respective population. We used descriptive statistics to summarise the fear ratings of participants, both at the sample level and by country, WHO region, economic development level, and COVID-19 severity level. Specifically, we categorised the countries based on their economic development levels as low, lower-middle, upper-middle, and high using the World Bank's 2020 report [15] and determined their COVID-19 severity categories (low, medium, and high) using tertiles of the average proportion of daily confirmed cases during the recruitment period from the WHO Coronavirus (COVID-19) Dashboard [16]. We used the multidimensional preference analysis (MDPREF) method for comparative analysis, which is ideal for visualising the relationships between fear factors and various factors, such as countries, regions, economic development level, and COVID-19 severity level. Specifically, MDPREF create biplots from ranking data, which position fear factors in a multidimensional space based on the perceived similarities or differences in fear rankings among countries or regions [17]. We determined the optimal number of dimensions for MDPREF using the scree plot method, identifying when additional factors did not substantially contribute to explaining variance. For our analysis, we weighted each country's fear factor data according to its age and gender distribution. Table S1 in the **Online Supplementary Document** provides details of the country classifications based on region, economic development, and COVID-19 pandemic severity. We performed the analyses in R, version 4.1.1 (R Core Team, Vienna, Austria).

## RESULTS

### Participants’ sociodemographic characteristics

We collected 19 145 responses, but 1940 were blank or 80% incomplete, 116 were duplicates, 450 were inconsistent, 126 were from countries outside of the 30 participating countries, and one did not provide age or gender data. We therefore included 16 512 responses in the final analysis. Most of these participants were women (62.7%) and the largest age group were the 18–24-year-olds (29.5%). Following weighting by age and gender of each country, we arrived at an overall sample size of 16 280 individuals, with 50.2% women (**Table 1**; Tables S2–3 in the **Online Supplementary Document**).

**Table 1:** Demographics and characteristics of 16 512 respondents*

Variables	Unweighted, n = 16 512	Weighted, n = 16 280
Gender		
*Female*	10 351 (62.7)	8171 (50.2)
*Male*	6061 (36.7)	8000 (49.1)
*Non-binary*	100 (0.6)	109 (0.7)
Age in years		
*18–24*	4857 (29.4)	1994 (12.3)
*25–29*	2345 (14.2)	1968 (12.1)
*30–34*	1931 (11.7)	1877 (11.5)
*35–39*	1855 (11.2)	1824 (11.2)
*40–44*	1427 (8.6)	1646 (10.1)
*45–49*	1157 (7.0)	1575 (9.7)
*50–54*	975 (5.9)	1388 (8.5)
*55–59*	667 (4.0)	1244 (7.6)
*60–64*	699 (4.2)	869 (5.3)
*≥65*	599 (3.6)	1894 (11.6)
Marital status		
*Married/cohabitation/common-law*	7275 (44.1)	9442 (58.0)
*Single*	8504 (51.5)	5645 (34.7)
*Separated/divorced/widowed*	732 (4.4)	1193 (7.3)
*Missing data*	1 (0.0)	1 (0.0)
Education		
*Primary or below*	405 (2.5)	729 (4.5)
*Secondary*	2627 (15.9)	2410 (14.8)
*Associate degree*	1576 (9.5)	1339 (8.2)
*Bachelor*	6500 (39.4)	5393 (33.1)
*College*	2258 (13.7)	2271 (13.9)
*Graduate*	2974 (18.0)	3976 (24.4)
*Missing*	172 (1.0)	162 (1.0)
Employment		
*Job seeking*	885 (5.4)	747 (4.6)
*Laid off*	170 (1.0)	197 (1.2)
*Not in workforce*	990 (6.0)	1233 (7.6)
*Retired*	614 (3.7)	1447 (8.9)
*Self-employed*	1309 (7.9)	1672 (10.3)
*Student*	4589 (27.8)	2103 (12.9)
*Working (≥40 h/week)*	5196 (31.5)	5683 (34.9)
*Working (1*–*39 h/week)*	2759 (16.7)	3198 (19.6)
BMI classification		
*Underweight*	1208 (19.5)	744 (4.5)
*Normal weight*	7456 (45.4)	6293 (38.7)
*Overweight*	3779 (23.0)	4315 (26.5)
*Obese*	3972 (24.2)	4797 (29.5)
*Missing data*	97 (0.6)	132 (0.8)
Pregnant		
*Yes*	226 (1.4)	283 (1.7)
*No*	10 179 (61.7)	7966 (48.9)
*Not applicable*	6107 (37.0)	8031 (49.3)
The need for regular medical follow-up before COVID-19		
*Yes*	4951 (30.0)	6117 (37.6)
*No*	11 558 (70.0)	10 160 (62.4)
*Missing data*	3 (0.0)	3 (0.0)
Practicing health professional		
*Yes*	4145 (25.1)	3922 (24.1)
*No*	12 366 (74.9)	12 358 (75.9)
*Missing data*	1 (0.0)	0 (0.0)
Having children less than 18 years of age		
*Yes*	4667 (28.3)	5369 (33.0)
*No*	11 845 (71.7)	10 911 (67.0)
Variables, x̄ (SD)		
*Perceived social rank, 1 = lowest to 5 = highest*	3.11 (0.9)	3.13 (0.92)
*Weight in kg*	65.62 (14.97)	68.45 (14.93)
*Height in m*	1.65 (0.09)	1.66 (0.10)
*BMI in kg/m2*	24.06 (4.70)	24.84 (4.71)
*Gestational week (if applicable)*	19.77 (12.9)	23.10 (14.16)
*Number of children less than 18 years old*	0.50 (0.96)	0.62 (1.08)
*Number of people in the household*	3.94 (2.04)	3.78 (2.01)
*House size in m^2^*	106.70 (107.55)	109.74 (107.72)

BMI – body mass index, SD – standard deviation, x̄ – mean

*Presented as n (%) unless specified otherwise.

### Participants’ fear of eleven factors

**Figure 1:**
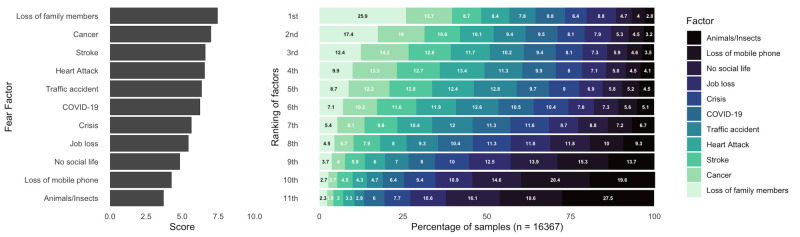
Respondents’ fear of eleven factors and proportion of those factors in each rank (n = 16 512).

**Figure 2:**
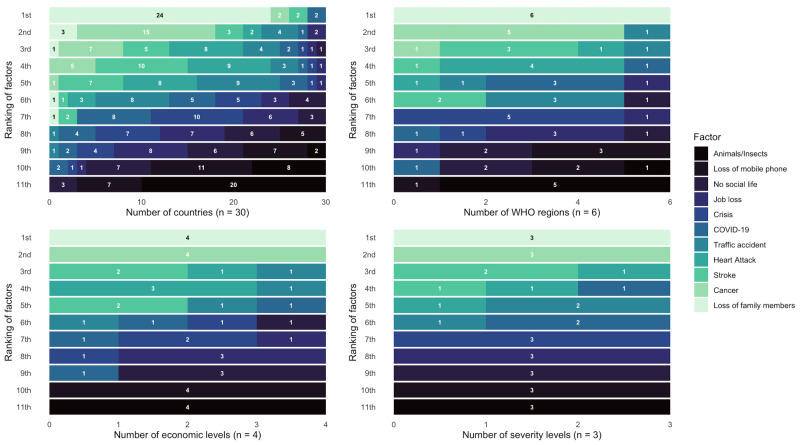
Proportion of 11 factors in each rank by country, WHO region, economic development level, and COVID-19 severity level.

Overall, based on the weighted mean fear score, the top three most feared factors were loss of family members (mean (x̄) = 7.46, standard deviation (SD) = 3.04), cancer (x̄ = 7.00, SD = 3.09), and stroke (x̄ = 6.61, SD = 3.24), while the three least feared factors were animals/insects (x̄ = 3.72, SD = 2.96), the loss of mobile phone (x̄ = 4.27, SD = 2.98), and no social life (x̄ = 4.83, SD = 3.13). Furthermore, the fear of COVID-19 was ranked sixth (x̄ = 6.23, SD = 2.92), in the middle of ranking (**Figure 1**; Table S4 in the **Online Supplementary Document**). Globally, 4232 (25.9%) participants ranked loss of family as their most fearful factor, followed by cancer (n = 2248, 13.7%), and stroke (n = 1416, 8.7%) (**Figure 2**). At the national level, 24 out of 30 countries rated the loss of family members as their top fear, while at all other levels (region, economic development level, and COVID-19 severity level), loss of family members was ranked first (Figure 2). Additional regional and country-level details are available in **Table 2**, **Figure 2**, and Figure S1 and Table S4 in the **Online Supplementary Document**.

**Table 2:** Weighted mean and standard deviation of fear of eleven factors by World Health Organization region, economic development level, and COVID-19 severity level*

Fear factors	WHO regions					Economic development levels						COVID-19 severity levels		
**EUR**	**AMR**	**EMR**	**WPR**	**AFR**		**SEAR**	**High**	**Upper-middle**	**Lower-middle**	**Low**	**High**	**Medium**	**Low**
Number of countries	3	6	5	9	4		3	12	9	6	3	10	10	10
Fear of factors (0–10, higher scores indicating higher level of fear)														
*1. COVID-19*	6.60 (2.48)	6.73 (2.93)	3.96 (3.00)	6.91 (2.49)	5.89 (3.16)		6.08 (2.59)	6.63 (2.68)	6.16 (2.98)	5.55 (2.99)	5.47 (3.42)	5.75 (3.18)	5.88 (2.97)	6.60 (2.72)
*2. Cancer*	7.78 (2.72)	7.29 (3.05)	5.62 (3.73)	7.55 (2.71)	6.62 (3.12)		6.36 (2.84)	7.42 (2.86)	6.90 (3.24)	6.26 (3.13)	6.53 (3.37)	6.85 (3.30)	6.75 (3.18)	7.19 (2.94)
*3. Stroke*	7.64 (2.98)	6.92 (3.41)	5.37 (3.76)	7.06 (2.91)	6.22 (3.12)		6.07 (2.94)	6.94 (3.10)	6.51 (3.40)	6.05 (3.16)	6.25 (3.36)	6.59 (3.54)	6.52 (3.24)	6.66 (3.10)
*4. Heart attack*	7.35 (2.94)	6.70 (3.34)	5.41 (3.71)	6.97 (2.91)	6.41 (3.08)		6.14 (2.93)	6.83 (3.08)	6.41 (3.32)	6.16 (3.18)	6.33 (3.27)	6.46 (3.43)	6.56 (3.23)	6.59 (3.07)
*5. Traffic accident*	6.53 (3.10)	6.31 (3.13)	5.33 (3.50)	6.67 (2.95)	6.46 (2.98)		6.45 (2.72)	6.42 (3.06)	6.33 (3.12)	6.13 (3.09)	6.52 (3.21)	6.03 (3.25)	6.43 (3.07)	6.45 (3.03)
*6. No social life*	5.61 (3.11)	3.97 (3.25)	4.66 (3.63)	5.22 (2.81)	4.81 (3.14)		4.55 (3.10)	5.24 (2.93)	4.02 (3.18)	4.80 (3.15)	5.86 (3.39)	4.24 (3.38)	4.97 (3.24)	5.02 (2.93)
*7. Crisis*	5.81 (3.52)	5.12 (3.46)	4.61 (3.65)	6.22 (3.20)	6.08 (3.22)		5.12 (3.11)	5.78 (3.39)	5.39 (3.40)	5.71 (3.27)	5.68 (3.50)	5.25 (3.46)	5.56 (3.46)	5.85 (3.30)
*8. Loss of family members*	8.12 (2.56)	7.63 (2.81)	6.59 (3.62)	7.79 (2.87)	7.09 (2.94)		7.15 (2.96)	7.53 (3.02)	7.57 (3.00)	7.15 (3.10)	7.21 (3.09)	7.36 (3.11)	7.65 (2.90)	7.40 (3.07)
*9. Animals/insects*	3.79 (3.10)	2.98 (2.85)	3.38 (3.31)	3.84 (2.85)	4.65 (2.98)		4.20 (2.59)	3.60 (2.90)	3.67 (2.99)	3.93 (2.83)	4.28 (3.42)	3.22 (3.00)	3.91 (3.02)	3.84 (2.89)
*10. Loss of mobile phone*	6.60 (2.48)	6.73 (2.93)	3.96 (3.00)	6.91 (2.49)	5.89 (3.16)		6.08 (2.59)	6.63 (2.68)	6.16 (2.98)	5.55 (2.99)	5.47 (3.42)	5.75 (3.18)	5.88 (2.97)	6.60 (2.72)
*11. Job loss*	7.78 (2.72)	7.29 (3.05)	5.62 (3.73)	7.55 (2.71)	6.62 (3.12)		6.36 (2.84)	7.42 (2.86)	6.90 (3.24)	6.26 (3.13)	6.53 (3.37)	6.85 (3.30)	6.75 (3.18)	7.19 (2.94)

AFR – African Region, AMR – Region of Americas, COVID-19 – coronavirus disease 2019, EMR – Eastern Mediterranean Region, EUR – European Region, SD – standard deviation, SEAR – South-East Asian Region, WHO – World Health Organization, WPR – Western Pacific Region, x̄ – mean

*Presented as x̄ (SD).

### Differences in fear ranking among countries and regions

**Figure 3:**
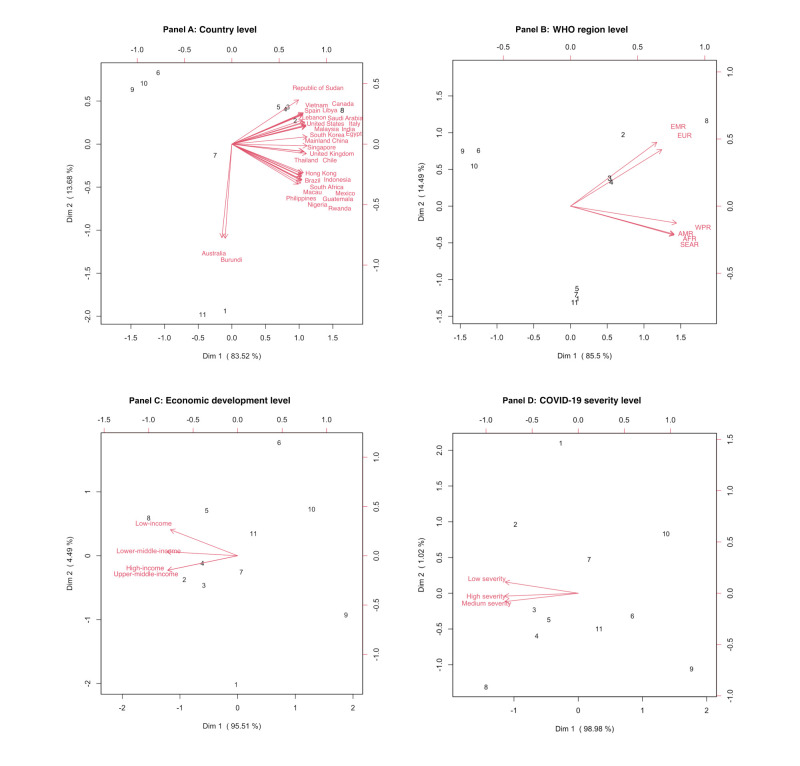
Biplots of multidimensional preference analysis, displayed as vectors for fear of factors and shown as numbers. **Panel A.** By country. **Panel B.** By WHO region. **Panel C.** By economic development level. **Panel D.** By COVID-19 severity level. The numbers in each panel refer to those listed in **Table 2**, while an arrow corresponds to a country or region and points toward increased level of fear. For each area, the projected length on the arrow corresponding to a particular country or region reflects the magnitude of the fear on that factor relative to other factors in the country. AFR – African Region, AMR – Region of Americas, EMR – Eastern Mediterranean Region, EUR – European Region, SEAR – South-East Asian Region, WPR – Western Pacific Region..

Based on the multidimensional preference analysis, at the country level (**Figure 3**, Panel A), people in Australia and Burundi most feared COVID-19 (#1) and job loss (#11), while people in other countries most feared the loss of family members (#8), followed by cancer (#2), stroke (#3), heart attack (#4), and traffic accident (#5). At the WHO region level (**Figure 3**, Panel B), economic development level (**Figure 3**, Panel C) and COVID-19 severity level (**Figure 3**, Panel D), loss of family members (#8) was the most feared factor for all objects, followed by cancer (#2), stroke (#3), and heart attack (#4). For all levels (**Figure 3**, Panels A–D), no social life (#6), animals/insects (#9), and loss of mobile phone (#10) were least feared factors.

## DISCUSSION

Our study provides a novel perspective on fear factors among diverse populations using representative international samples, with significant implications for public health policy. Specifically, we identified the loss of family members as the most feared event across diverse populations, followed by cancer as the most feared non-communicable disease. Notably, non-communicable diseases were more feared than COVID-19. While most countries had similar fear rankings, prioritising loss of family members and non-communicable disease like cancer, stroke, and heart attack, we found notable exceptions in Australia and Burundi, where COVID-19 and job loss were the dominant fears. This pattern, consistent across various WHO regions, economic levels, and COVID-19 severity categories, suggests a regional trend in fear perceptions. However, the marked variations in specific countries underscore the importance of tailoring public health strategies to local contexts and needs. Overall, these findings could inform policy decisions in view of identifying the events that require attention to alleviate fear; highlighting the areas that need medical service improvements; providing suggestions for effective public health messaging; improving public health emergency management; guiding resource allocation; and promoting global collaboration.

This study provides a unique finding that losing a family member is the most prominent source of fear across diverse population groups. This fear may arise from associating the loss with the negative impacts of bereavement – an intensely personal and emotional experience that can have long-lasting impacts on one's well-being, affecting over 30 aspects of well-being, including affective, cognitive, behavioural, physiological-somatic, immunological, and endocrine changes such as depression, suicidal ideation, social withdrawal, sleep disturbance, somatic complaints, and susceptibility to illness, disease, and mortality [18]. Given the significant impact of bereavement, health care practitioners must recognise and address its lasting effects. Unfortunately, bereavement support is often not given enough attention, even in settings that provide palliative care [19]. Therefore, health care practitioners should focus their efforts on assessing and supporting family caregivers during the pre-bereavement period while developing community capacity and referral pathways for bereavement care. A compassionate and empathetic approach should be provided for not only at-risk dying patients, but also for their family members. Public health strategies should include bereavement support programs and initiatives to educate the public on coping mechanisms for loss, while mental health resources should be made more accessible, particularly in communities with high mortality rates. Moreover, sudden death can cause more severe and prolonged physical stress to bereaved individuals than death from chronic diseases [20], suggesting that it is imperative for policymakers to prioritise prevention and early detection of diseases that may contribute to premature or sudden loss of life. This includes diseases that place individuals at risk to die at a young age and early stage, such as sudden infant death syndrome, aortic dissection, sudden unexpected death in epilepsy, and cardiac arrhythmia, to reduce the occurrence of uncommon bereavement. Aligning public health messaging with people’s concerns, such as the fear of losing loved ones, can make messaging more relevant and effective. By communicating how certain behaviours like vaccination or healthy lifestyle choices can protect family members, public health campaigns can resonate more deeply with the population's concerns without exacerbating their fears. Dyadic interventions designed to enhance patients' health outcomes can also be effective by evoking the profound fear their family members experience at the thought of losing them.

One notable finding is that cancer was the most feared non-communicable disease, surpassing heart disease and stroke, which are typically identified as the leading causes of death globally [21]. This fear is rooted in the belief that cancer is a vicious, unpredictable, and unbeatable enemy that can cause significant physical, emotional, and financial burdens on patients and their families [22]. Addressing this fear is crucial for medical practitioners both before and after a cancer diagnosis. Before the diagnosis, it can lead to avoidance behaviour and hinder the uptake of cancer screening and early treatment [7]. To address this, public health strategies should emphasise education and awareness campaigns to promote early cancer screening and debunk myths surrounding the disease. Additionally, fear can also impact cancer patients' adherence to treatment, prognostic outcomes, and quality of life [23]. Thus, enhancing support services for emotional and psychological care can improve treatment adherence and quality of life for cancer patients. Moreover, the mass media shapes public perceptions and attitudes towards cancer and bears certain responsibility for past stigmatisation and fear of cancer [24]. Policymakers can therefore ensure mass media provides accurate and balanced information about cancer and fund cancer education programs to promote critical thinking and informed decision-making among the general population. After the diagnoses, individuals will also benefit from prioritizing cancer research and investing in emotional support for themselves and their families.

Another significant finding of our study is that people tended to exhibit less fear towards infectious diseases such as COVID-19 in comparison to non-communicable diseases such as cancer, stroke, and heart attack. This is an important observation, given that our study was conducted during the circulation of Alpha, Beta, and Delta variants of the severe acute respiratory syndrome coronavirus 2 (SARS-CoV-2), which had a slightly higher case-fatality ratio of an average of 1.02% worldwide as of March 2023 [16]. One possible explanation for this fear ranking could be the comparatively lower fatality rate of COVID-19 in comparison to non-communicable diseases, which can have severe long-term impacts on health and well-being. These findings could provide valuable insights for future pandemics, where data from COVID-19 can be used as a benchmark to predict the fear of infectious diseases among the population. This, in turn, can help governments balance between over-dissemination (leading to panic) and under-dissemination (leading to indifference to prevent measures) among community residents by adjusting the media coverage of the disease. Furthermore, our study emphasises the importance of prioritising prevention, diagnosis, and treatment of non-communicable diseases during public health emergencies. Efforts to balance public perception through education about the risks of non-communicable diseases and infectious diseases are needed. Public health messaging should equally emphasise the importance of managing chronic diseases even during pandemics.

We also observed a similar trend in fear ranking across many countries, WHO regions, economic development levels, and COVID-19 severity levels. However, the distinct priorities observed in countries like Australia and Burundi, where COVID-19 and job loss were most feared, underscore the influence of specific cultural, economic, and regional factors on fear perceptions. In Australia, an intensified fear of COVID-19 emerged from cultural traits such as short-term orientation, individualism, and indulgence [25], further amplified by stringent pandemic control measures [26]. This cultural perspective led Australians to view the pandemic more as a widespread socioeconomic threat than merely a health issue, a viewpoint underscored by significant economic impacts, including a 15% job loss rate [27,28]. In a culture that values individualism and indulgence, potentially combined with limited financial savings, these factors collectively heightened fears surrounding job loss. Conversely, in Burundi, the acute fear of COVID-19 is rooted in extreme poverty and critically inadequate medical resources, evidenced by one of the lowest physician-to-population ratios globally (approximately three per 100 000 people) [29]. The country's lower awareness of diseases, especially non-communicable ones like cancer, likely led to an underestimation of these health risks [29]. Furthermore, the deaths of national leaders from COVID-19 may have intensified the focus on the immediate threat posed by the virus [30,31]. Additionally, in the context of Burundi, where job loss could be equated to a life-or-death struggle due to extreme poverty, economic fears are as significant as health concerns. This explains why the fear of job loss is also prevalent in the country. In Australia and Burundi, addressing job loss fears through financial support is crucial. Specifically, for COVID-19 fears, Australia's response should target its social impacts, while Burundi needs to bolster health infrastructure and manage economic insecuritie*s.*

As this study was conducted during the COVID-19 pandemic, we must consider the pandemic's impact on participants' responses and the interpretation of results. The pandemic likely heightened fears of infectious diseases, especially COVID-19, and the fear of losing loved ones due to the observed mortality from COVID-19 [32,33]. For non-communicable diseases like cancer, heart attack, and stroke, the impact was dual. While COVID-19's focus might have temporarily reduced these diseases' immediate fear, delays in medical screenings and treatments could have increased fears of late diagnoses and poor outcomes [6,34]. Conversely, fears unrelated to health, such as traffic accidents or fear of insects, might have diminished as public attention focused on the pandemic. The pandemic's varied impact on different fears underscores the need for future studies in non-pandemic contexts to determine if these fear rankings are consistent.

### Limitations

Our study, while informative, has several limitations. First, the data collection was primarily conducted online using convenience sampling method, which may limit representation of individuals with low socio-economic status, limited digital literacy, or inadequate access to the internet or digital devices. While we have attempted to mitigate this issue by weighting our samples according to the demographics of the corresponding populations, there remains an inherent selection bias. Consequently, this bias may limit the generalisability of our findings across the entire population. Future studies should consider addressing this by employing stratified sampling methods and integrating offline data collection to better represent individuals with limited internet access. Second, our study's cross-sectional design inherently limits our ability to establish causality or track how fears evolve over time or in response to events such as a new pandemic. Future studies should therefore adopt a longitudinal design to better understand these dynamic changes, as it would allow for the observation of fear perceptions as they develop and respond to changing circumstances. Such studies would provide a more nuanced understanding of the temporal dynamics of fear and its causal factors. Third, the study's timing during the COVID-19 pandemic may have heightened fears of infectious diseases and losing loved ones, while reducing concerns over non-health issues. Future non-pandemic research is crucial to validate these fear rankings. Fourth, our quantitative methodology may have limitations in capturing the nuanced and subjective aspects of fear. Future research could address this through qualitative approaches. Finally, while we effectively identified the fear ranking, we did not explore the underlying factors associated with this ranking. Future research should employ qualitative or mixed-methods approach to explore the contextual and cultural determinants shaping these rankings more comprehensively.

## CONCLUSIONS

Adopting effective measures to address fear is crucial in promoting effective health messaging and mitigating the adverse impacts on individuals and health care systems. Healthcare campaigns should prioritise allocating additional resources to tackle top-ranked factors, including the loss of family members, cancer, and stroke. In particular, the fear of losing family members underscores the importance of promoting bereavement care and preventing diseases that can lead to early or sudden death, especially in the context of the COVID-19 pandemic and its impact on global mortality rates. Public health messages that align with these concerns, emphasising protective behaviours, can effectively motivate the public without causing undue fear. Urgent attention should also be given to reducing cancer fear-based avoidance behaviours towards prevention services, improving early detection and treatment, and enhancing prognostic outcomes. In public health emergencies, the prevention, diagnosis, and treatment of non-communicable diseases should not be ignored. Burundi and Australia need to address their unique fears related to COVID-19 and job loss currently. Future studies are expected to examine the uniqueness of their fear rankings during non-pandemic times. Global collaborations are encouraged to resolve the issue of perceived fear among countries and regions that shared similar fear rankings.


Online Supplementary Document.
