# Supplementary material for: Fear in general populations: A cross-sectional study on perceived fear of common diseases, COVID-19, life events, and environmental threats in 30 countries
Source: J Glob Health. 2024 Jun 7;14:05019. doi: 10.7189/jogh.14.05019 (PMC11156250; doi:10.7189/jogh.14.05019)

ONLINE SUPPLEMENTARY DOCUMENT

Title: Fear in general populations: A cross-sectional study on perceived fear of common diseases, COVID-19, life events, and environmental threats in 30 countries

Authors: Jiaying Li, Vinciya Pandian, Daniel Yee Tak Fong, Kris Yuet Wan Lok, Janet Yuen Ha Wong, Mandy Man Ho, Edmond Pui Hang Choi, Patricia M Davidson, Wenjie Duan, Marie Tarrant, Jung Jae Lee, Chia-Chin Lin, Oluwadamilare Akingbade, Khalid M Alabdulwahhab, Mohammad Shakil Ahmad, Mohamed Alboraie, Meshari A Alzahrani, Anil S Bilimale, Sawitree Boonpatcharanon, Samuel Byiringiro, Muhammad Kamil Che Hasan, Luisa Clausi Schettini, Walter Corzo, Josephine M. De Leon, Anjanette S. De Leon, Hiba Deek, Fabio Efficace, Mayssah A El Nayal, Fathiya El-Raey, Eduardo Ensaldo-Carrasco, Pilar Escotorin, Oluwadamilola Agnes Fadodun, Israel Opeyemi Fawole, Yong-Shian Shawn Goh, Devi Irawan, Naimah Ebrahim Khan, Binu Koirala, Ashish Krishna, Cannas Kwok, Tung Thanh Le, Daniela Giambruno Leal, Miguel Ángel Lezana-Fernández, Emery Manirambona, Leandro Cruz Mantoani, Fernando Meneses-González, Iman Elmahdi Mohamed, Madeleine Mukeshimana, Chinh Thi Minh Nguyen, Huong Thi Thanh Nguyen, Khanh Thi Nguyen, Son Truong Nguyen, Mohd Said Nurumal, Aimable Nzabonimana, Nagla Abdelrahim Mohamed Ahmed Omer, Oluwabunmi Ogungbe, Angela Chiu Yin Poon, Areli Reséndiz-Rodriguez, Busayasachee Puang-Ngern, Ceryl G Sagun, Riyaz Ahmed Shaik, Nikhil Gauri Shankar, Kathrin Sommer, Edgardo Toro, Hanh Thi Hong Tran, Elvira L Urgel, Emmanuel Uwiringiyimana, Tita Vanichbuncha, Naglaa Youssef

Supplementary material summary

**Contents**	**Numbered pages**
eTable 1. World Health Organization (WHO) regions, economic development levels and COVID-19 severity levels of the 30 countries or regions	2
eTable 2. Demographic summary by country	3-10
eTable 3. Weighted demographic summary by country	11-18
eTable 4. Description of respondents' weighted fear of 11 factors by country.	19-20
eFigure 1. Visualization of the weighted fear of 11 factors by country, World Health Organization (WHO) region, economic development level, and COVID-19 severity level.	21-29

eTable 1. World Health Organization (WHO) regions, economic development levels and COVID-19 severity levels of the 30 countries or regions.

Country	Region	Economic development level	COVID-19 severity level
Australia	Western Pacific Region	High-income	Low severity
Brazil	Region of Americas	Upper-middle-income	Medium severity
Burundi	African Region	Low-income	Low severity
Canada	Region of Americas	High-income	High severity
Chile	Region of Americas	High-income	High severity
Egypt	Eastern Mediterranean Region	Lower-middle-income	Low severity
Guatemala	Region of Americas	Upper-middle-income	Medium severity
Hong Kong	Western Pacific Region	High-income	Low severity
India	South-East Asian Region	Lower-middle-income	Medium severity
Indonesia	South-East Asian Region	Lower-middle-income	Medium severity
Italy	European Region	High-income	High severity
Lebanon	Eastern Mediterranean Region	Upper-middle-income	High severity
Libya	Eastern Mediterranean Region	Upper-middle-income	High severity
Macau	Western Pacific Region	High-income	Low severity
Mainland China	Western Pacific Region	Upper-middle-income	Low severity
Malaysia	Western Pacific Region	Upper-middle-income	Medium severity
Mexico	Region of Americas	Upper-middle-income	High severity
Nigeria	African Region	Lower-middle-income	Low severity
Philippines	Western Pacific Region	Lower-middle-income	Medium severity
Republic of Sudan	Eastern Mediterranean Region	Low-income	Medium severity
Rwanda	African Region	Low-income	Medium severity
Saudi Arabia	Eastern Mediterranean Region	High-income	Medium severity
Singapore	Western Pacific Region	High-income	Medium severity
South African Regionica	African Region	Upper-middle-income	High severity
South Korea	Western Pacific Region	High-income	Low severity
Spain	European Region	High-income	High severity
Thailand	South-East Asian Region	Upper-middle-income	Low severity
United Kingdom	European Region	High-income	High severity
United States	Region of Americas	High-income	High severity
Vietnam	Western Pacific Region	Lower-middle-income	Low severity

eTable 2. Demographic summary by country.

Characteristic	Australia, N = 639[1]	Brazil, N = 553[1]	Burundi, N = 369[1]	Canada, N = 368[1]	Chile, N = 342[1]	Egypt, N = 461[1]	Guatemala, N = 229[1]	Hong Kong, N = 2,127[1]	India, N = 529[1]	Indonesia, N = 482[1]	Italy, N = 203[1]	Lebanon, N = 440[1]	Libya, N = 645[1]	Macau, N = 250[1]	Mainland China, N = 667[1]
Age															
18-24	106 (17%)	128 (23%)	41 (11%)	20 (5·4%)	75 (22%)	103 (22%)	88 (38%)	196 (9·2%)	151 (29%)	433 (90%)	4 (2·0%)	210 (48%)	246 (38%)	70 (28%)	477 (72%)
25-29	84 (13%)	95 (17%)	44 (12%)	35 (9·5%)	45 (13%)	71 (15%)	43 (19%)	239 (11%)	98 (19%)	23 (4·8%)	12 (5·9%)	44 (10%)	138 (21%)	31 (12%)	94 (14%)
30-34	32 (5·0%)	73 (13%)	70 (19%)	48 (13%)	32 (9·4%)	85 (18%)	27 (12%)	315 (15%)	53 (10%)	11 (2·3%)	14 (6·9%)	53 (12%)	92 (14%)	43 (17%)	29 (4·3%)
35-39	49 (7·7%)	95 (17%)	77 (21%)	52 (14%)	40 (12%)	87 (19%)	22 (9·6%)	328 (15%)	94 (18%)	6 (1·2%)	16 (7·9%)	51 (12%)	70 (11%)	41 (16%)	15 (2·2%)
40-44	45 (7·0%)	41 (7·4%)	70 (19%)	33 (9·0%)	44 (13%)	52 (11%)	12 (5·2%)	240 (11%)	40 (7·6%)	2 (0·4%)	25 (12%)	29 (6·6%)	40 (6·2%)	27 (11%)	15 (2·2%)
45-49	80 (13%)	26 (4·7%)	32 (8·7%)	14 (3·8%)	43 (13%)	27 (5·9%)	18 (7·9%)	253 (12%)	31 (5·9%)	2 (0·4%)	29 (14%)	16 (3·6%)	22 (3·4%)	13 (5·2%)	25 (3·7%)
50-54	64 (10%)	28 (5·1%)	16 (4·3%)	26 (7·1%)	33 (9·6%)	22 (4·8%)	7 (3·1%)	165 (7·8%)	11 (2·1%)	3 (0·6%)	19 (9·4%)	16 (3·6%)	22 (3·4%)	11 (4·4%)	8 (1·2%)
55-59	69 (11%)	25 (4·5%)	9 (2·4%)	35 (9·5%)	12 (3·5%)	8 (1·7%)	6 (2·6%)	117 (5·5%)	17 (3·2%)	2 (0·4%)	36 (18%)	13 (3·0%)	10 (1·6%)	5 (2·0%)	4 (0·6%)
60-64	14 (2·2%)	25 (4·5%)	5 (1·4%)	49 (13%)	8 (2·3%)	4 (0·9%)	3 (1·3%)	125 (5·9%)	24 (4·5%)	0 (0%)	8 (3·9%)	6 (1·4%)	4 (0·6%)	8 (3·2%)	0 (0%)
>=65	96 (15%)	17 (3·1%)	5 (1·4%)	56 (15%)	10 (2·9%)	2 (0·4%)	3 (1·3%)	149 (7·0%)	10 (1·9%)	0 (0%)	40 (20%)	2 (0·5%)	1 (0·2%)	1 (0·4%)	0 (0%)
Gender															
Female	376 (59%)	395 (71%)	181 (49%)	297 (81%)	251 (73%)	266 (58%)	119 (52%)	1,186 (56%)	269 (51%)	396 (82%)	133 (66%)	291 (66%)	376 (58%)	201 (80%)	523 (78%)
Male	260 (41%)	157 (28%)	177 (48%)	64 (17%)	84 (25%)	195 (42%)	107 (47%)	940 (44%)	260 (49%)	86 (18%)	70 (34%)	149 (34%)	269 (42%)	49 (20%)	140 (21%)
Non-binary	3 (0·5%)	1 (0·2%)	11 (3·0%)	7 (1·9%)	7 (2·0%)	0 (0%)	3 (1·3%)	1 (<0·1%)	0 (0%)	0 (0%)	0 (0%)	0 (0%)	0 (0%)	0 (0%)	4 (0·6%)
Marital															
Married/Cohabitation/Common-law	332 (52%)	256 (46%)	236 (64%)	256 (70%)	120 (35%)	274 (59%)	71 (31%)	1,330 (63%)	275 (52%)	42 (8·7%)	145 (71%)	172 (39%)	211 (33%)	123 (49%)	113 (17%)
Separated/Divorced/Widowed	91 (14%)	39 (7·1%)	14 (3·8%)	40 (11%)	39 (11%)	17 (3·7%)	3 (1·3%)	99 (4·7%)	28 (5·3%)	0 (0%)	24 (12%)	12 (2·7%)	15 (2·3%)	5 (2·0%)	8 (1·2%)
Single	215 (34%)	258 (47%)	119 (32%)	72 (20%)	183 (54%)	170 (37%)	155 (68%)	698 (33%)	226 (43%)	440 (91%)	34 (17%)	256 (58%)	419 (65%)	122 (49%)	546 (82%)
Unknown	1 (0·2%)	0 (0%)	0 (0%)	0 (0%)	0 (0%)	0 (0%)	0 (0%)	0 (0%)	0 (0%)	0 (0%)	0 (0%)	0 (0%)	0 (0%)	0 (0%)	0 (0%)
Education															
Primary or below	0 (0%)	1 (0·2%)	139 (38%)	1 (0·3%)	3 (0·9%)	1 (0·2%)	0 (0%)	167 (7·9%)	42 (7·9%)	0 (0%)	3 (1·5%)	6 (1·4%)	3 (0·5%)	0 (0%)	2 (0·3%)
Secondary	156 (24%)	61 (11%)	180 (49%)	44 (12%)	68 (20%)	28 (6·1%)	49 (21%)	1,123 (53%)	61 (12%)	0 (0%)	6 (3·0%)	49 (11%)	96 (15%)	24 (9·6%)	59 (8·8%)
College	195 (31%)	52 (9·4%)	16 (4·3%)	103 (28%)	124 (36%)	33 (7·2%)	71 (31%)	181 (8·5%)	43 (8·1%)	91 (19%)	12 (5·9%)	27 (6·1%)	68 (11%)	4 (1·6%)	103 (15%)
Associate degree	130 (20%)	9 (1·6%)	6 (1·6%)	7 (1·9%)	8 (2·3%)	23 (5·0%)	2 (0·9%)	196 (9·2%)	40 (7·6%)	170 (35%)	63 (31%)	73 (17%)	92 (14%)	158 (63%)	8 (1·2%)

Bachelor	76 (12%)	154 (28%)	24 (6·5%)	126 (34%)	51 (15%)	230 (50%)	45 (20%)	415 (20%)	142 (27%)	208 (43%)	20 (9·9%)	199 (45%)	291 (45%)	51 (20%)	350 (52%)
Graduate	43 (6·7%)	276 (50%)	4 (1·1%)	87 (24%)	88 (26%)	146 (32%)	62 (27%)	45 (2·1%)	197 (37%)	13 (2·7%)	99 (49%)	86 (20%)	95 (15%)	13 (5·2%)	140 (21%)
Unkown	39 (6·1%)	0 (0%)	0 (0%)	0 (0%)	0 (0%)	0 (0%)	0 (0%)	0 (0%)	4 (0·8%)	0 (0%)	0 (0%)	0 (0%)	0 (0%)	0 (0%)	5 (0·7%)
Employment															
Job seeking	74 (12%)	10 (1·8%)	16 (4·3%)	19 (5·2%)	23 (6·7%)	24 (5·2%)	6 (2·6%)	69 (3·2%)	29 (5·5%)	5 (1·0%)	6 (3·0%)	60 (14%)	48 (7·4%)	3 (1·2%)	55 (8·2%)
Laid off	53 (8·3%)	1 (0·2%)	11 (3·0%)	10 (2·7%)	0 (0%)	0 (0%)	2 (0·9%)	45 (2·1%)	1 (0·2%)	0 (0%)	0 (0%)	5 (1·1%)	2 (0·3%)	0 (0%)	4 (0·6%)
Not in workforce	34 (5·3%)	8 (1·4%)	4 (1·1%)	50 (14%)	14 (4·1%)	46 (10·0%)	0 (0%)	214 (10%)	59 (11%)	2 (0·4%)	9 (4·4%)	34 (7·7%)	25 (3·9%)	4 (1·6%)	7 (1·0%)
Retired	41 (6·4%)	31 (5·6%)	3 (0·8%)	80 (22%)	12 (3·5%)	7 (1·5%)	1 (0·4%)	185 (8·7%)	12 (2·3%)	0 (0%)	45 (22%)	2 (0·5%)	2 (0·3%)	2 (0·8%)	2 (0·3%)
Self-employed	4 (0·6%)	62 (11%)	215 (58%)	28 (7·6%)	18 (5·3%)	50 (11%)	23 (10%)	34 (1·6%)	57 (11%)	11 (2·3%)	26 (13%)	39 (8·9%)	51 (7·9%)	13 (5·2%)	7 (1·0%)
Student	47 (7·4%)	130 (24%)	58 (16%)	26 (7·1%)	88 (26%)	89 (19%)	99 (43%)	162 (7·6%)	159 (30%)	417 (87%)	6 (3·0%)	149 (34%)	265 (41%)	57 (23%)	383 (57%)
Working (>=40hrs/wk)	58 (9·1%)	210 (38%)	21 (5·7%)	85 (23%)	153 (45%)	136 (30%)	65 (28%)	1,082 (51%)	153 (29%)	27 (5·6%)	55 (27%)	69 (16%)	75 (12%)	87 (35%)	128 (19%)
Working (1-39hrs/wk)	328 (51%)	101 (18%)	41 (11%)	70 (19%)	34 (9·9%)	109 (24%)	33 (14%)	336 (16%)	59 (11%)	20 (4·1%)	56 (28%)	82 (19%)	177 (27%)	84 (34%)	81 (12%)
Height (m)	1·69 (0·08) (1·50 to 1·96)	1·67 (0·10) (1·16 to 1·94)	1·63 (0·05) (1·52 to 1·90)	1·67 (0·10) (1·28 to 2·11)	1·64 (0·08) (1·40 to 1·90)	1·68 (0·10) (1·30 to 1·97)	1·66 (0·10) (1·44 to 1·98)	1·64 (0·08) (1·37 to 1·98)	1·64 (0·10) (1·43 to 1·96)	1·60 (0·06) (1·45 to 1·84)	1·69 (0·08) (1·50 to 1·94)	1·67 (0·09) (1·47 to 1·98)	1·67 (0·11) (1·20 to 2·00)	1·62 (0·08) (1·49 to 1·98)	1·64 (0·07) (1·30 to 1·86)
Unknown	12	0	1	5	0	0	2	1	4	0	0	3	2	0	2
Weight(kg)	72 (14) (27 to 130)	70 (16) (37 to 120)	60 (6) (45 to 89)	75 (18) (31 to 127)	71 (14) (41 to 113)	80 (18) (40 to 160)	68 (14) (39 to 110)	62 (10) (39 to 117)	66 (13) (34 to 104)	55 (11) (33 to 103)	70 (15) (45 to 122)	70 (16) (40 to 140)	71 (17) (36 to 150)	58 (13) (30 to 110)	61 (17) (35 to 135)
Unknown	9	0	0	8	0	0	2	0	4	0	0	0	0	0	2
BMI	25·2 (4·1) (11·7 to 45·8)	25·2 (4·7) (15·6 to 42·0)	22·5 (1·9) (15·5 to 34·0)	26·8 (5·6) (13·5 to 43·8)	26·3 (4·2) (17·6 to 44·4)	28·2 (5·6) (15·4 to 62·4)	24·4 (4·1) (13·9 to 37·1)	22·8 (3·0) (13·4 to 38·2)	24·4 (3·9) (14·5 to 36·1)	21·4 (3·9) (14·1 to 40·3)	24·7 (4·4) (17·3 to 38·9)	25·0 (4·7) (13·8 to 48·4)	25·6 (5·6) (12·9 to 52·1)	21·9 (4·0) (11·0 to 37·5)	22·4 (5·8) (12·1 to 48·4)
Unknown	15	0	1	9	0	0	4	1	5	0	0	3	2	0	2
BMI classification															
Severely underweight	7 (1·1%)	1 (0·2%)	2 (0·5%)	2 (0·6%)	0 (0%)	1 (0·2%)	4 (1·8%)	9 (0·4%)	6 (1·1%)	18 (3·7%)	0 (0%)	1 (0·2%)	11 (1·7%)	10 (4·0%)	15 (2·3%)
Underweight	17 (2·7%)	17 (3·1%)	2 (0·5%)	7 (1·9%)	4 (1·2%)	8 (1·7%)	4 (1·8%)	126 (5·9%)	21 (4·0%)	90 (19%)	4 (2·0%)	21 (4·8%)	22 (3·4%)	38 (15%)	100 (15%)
Normal weight	281 (45%)	280 (51%)	344 (93%)	142 (40%)	132 (39%)	68 (15%)	123 (55%)	925 (44%)	174 (33%)	240 (50%)	118 (58%)	148 (34%)	297 (46%)	120 (48%)	356 (54%)
Overweight	241 (39%)	171 (31%)	15 (4·1%)	114 (32%)	143 (42%)	59 (13%)	72 (32%)	620 (29%)	91 (17%)	58 (12%)	58 (29%)	62 (14%)	201 (31%)	32 (13%)	68 (10%)
Obesity	78 (12%)	84 (15%)	5 (1·4%)	94 (26%)	63 (18%)	325 (70%)	22 (9·8%)	446 (21%)	232 (44%)	76 (16%)	23 (11%)	205 (47%)	112 (17%)	50 (20%)	126 (19%)

Unknown	15	0	1	9	0	0	4	1	5	0	0	3	2	0	2
Pregnant															
Not applicable	261 (41%)	157 (28%)	177 (48%)	64 (17%)	84 (25%)	219 (48%)	107 (47%)	940 (44%)	260 (49%)	86 (18%)	71 (35%)	159 (36%)	269 (42%)	49 (20%)	141 (21%)
No	372 (58%)	393 (71%)	159 (43%)	280 (76%)	256 (75%)	242 (52%)	119 (52%)	1,177 (55%)	268 (51%)	390 (81%)	131 (65%)	277 (63%)	357 (55%)	189 (76%)	506 (76%)
Yes	6 (0·9%)	3 (0·5%)	33 (8·9%)	24 (6·5%)	2 (0·6%)	0 (0%)	3 (1·3%)	10 (0·5%)	1 (0·2%)	6 (1·2%)	1 (0·5%)	4 (0·9%)	19 (2·9%)	12 (4·8%)	20 (3·0%)
Gestational week	22 (13) (6 to 40)	6 (1) (6 to 7)	4 (1) (3 to 7)	24 (12) (4 to 39)	30 (NA) (30 to 30)	NA (NA) (Inf to -Inf)	NA (NA) (Inf to -Inf)	24 (9) (15 to 40)	NA (NA) (Inf to -Inf)	19 (12) (8 to 32)	12 (NA) (12 to 12)	16 (12) (6 to 30)	22 (12) (9 to 36)	30 (13) (5 to 40)	30 (12) (4 to 41)
Unknown	633	551	348	345	341	461	229	2,117	529	477	202	436	638	238	655
Regular medical follow-up before COVID-19															
NA	1 (0·2%)	0 (0%)	0 (0%)	0 (0%)	0 (0%)	0 (0%)	0 (0%)	0 (0%)	0 (0%)	0 (0%)	0 (0%)	0 (0%)	0 (0%)	0 (0%)	0 (0%)
No	475 (74%)	393 (71%)	352 (95%)	119 (32%)	207 (61%)	347 (75%)	170 (74%)	1,606 (76%)	377 (71%)	365 (76%)	44 (22%)	287 (65%)	450 (70%)	199 (80%)	628 (94%)
Yes	163 (26%)	160 (29%)	17 (4·6%)	249 (68%)	135 (39%)	114 (25%)	59 (26%)	521 (24%)	152 (29%)	117 (24%)	159 (78%)	153 (35%)	195 (30%)	51 (20%)	39 (5·8%)
Practising healthcare professional															
NA	0 (0%)	0 (0%)	0 (0%)	0 (0%)	0 (0%)	0 (0%)	0 (0%)	0 (0%)	0 (0%)	1 (0·2%)	0 (0%)	0 (0%)	0 (0%)	0 (0%)	0 (0%)
No	563 (88%)	339 (61%)	356 (96%)	332 (90%)	303 (89%)	253 (55%)	177 (77%)	2,068 (97%)	342 (65%)	261 (54%)	184 (91%)	261 (59%)	427 (66%)	148 (59%)	645 (97%)
Yes	76 (12%)	214 (39%)	13 (3·5%)	36 (9·8%)	39 (11%)	208 (45%)	52 (23%)	59 (2·8%)	187 (35%)	220 (46%)	19 (9·4%)	179 (41%)	218 (34%)	102 (41%)	22 (3·3%)
Number of children less than 18 years old															
No	513 (80%)	410 (74%)	153 (41%)	242 (66%)	220 (64%)	215 (47%)	182 (79%)	1,518 (71%)	328 (62%)	465 (96%)	145 (71%)	307 (70%)	474 (73%)	132 (53%)	383 (57%)
Yes	126 (20%)	143 (26%)	216 (59%)	126 (34%)	122 (36%)	246 (53%)	47 (21%)	609 (29%)	201 (38%)	17 (3·5%)	58 (29%)	133 (30%)	171 (27%)	118 (47%)	284 (43%)
Number of children aged above 18	0·34 (0·74) (0·00 to 3·00)	0·40 (0·82) (0·00 to 8·00)	1·25 (1·21) (0·00 to 6·00)	0·61 (1·01) (0·00 to 5·00)	0·55 (0·88) (0·00 to 5·00)	1·13 (1·27) (0·00 to 5·00)	0·32 (0·74) (0·00 to 5·00)	0·39 (0·68) (0·00 to 4·00)	0·59 (0·86) (0·00 to 5·00)	0·04 (0·20) (0·00 to 2·00)	0·47 (0·83) (0·00 to 3·00)	0·69 (1·17) (0·00 to 5·00)	0·71 (1·39) (0·00 to 6·00)	0·80 (1·05) (0·00 to 7·00)	0·54 (0·75) (0·00 to 6·00)
Unknown	0	7	1	3	3	9	3	0	1	0	0	0	11	1	7
Number of people lived with	2·90 (1·19) (1·00 to 6·00)	2·89 (1·29) (1·00 to 7·00)	4·08 (1·31) (1·00 to 8·00)	2·61 (1·37) (1·00 to 7·00)	3·26 (1·44) (1·00 to 8·00)	4·67 (1·83) (1·00 to 17·00)	4·52 (2·25) (1·00 to 12·00)	3·35 (1·08) (1·00 to 9·00)	3·86 (1·72) (1·00 to 20·00)	4·35 (1·30) (1·00 to 9·00)	2·50 (1·17) (1·00 to 6·00)	4·60 (1·54) (1·00 to 11·00)	6·19 (2·88) (1·00 to 26·00)	3·84 (1·45) (1·00 to 12·00)	3·75 (1·39) (1·00 to 10·00)
Unknown	51	0	2	2	0	4	0	0	2	0	2	0	21	0	12
Perceived social rank	3·13 (0·93) (1·00 to	3·42 (0·79) (1·00 to	1·66 (0·68) (1·00 to	3·46 (0·93) (1·00 to	3·12 (0·77) (1·00 to	3·32 (0·91) (1·00 to	3·15 (0·75) (1·00 to	2·73 (0·76) (1·00 to	3·18 (1·08) (1·00 to	3·51 (0·87) (1·00 to	3·60 (0·69) (2·00 to	3·03 (0·81) (1·00 to	3·21 (1·11) (1·00 to	3·21 (0·70) (1·00 to	2·77 (0·77) (1·00 to

	5·00)	5·00)	4·00)	5·00)	5·00)	5·00)	5·00)	5·00)	5·00)	5·00)	5·00)	5·00)	5·00)	5·00)	5·00)
Unknown	1	0	0	0	0	0	0	0	1	1	0	0	0	0	1
House size	175 (138) (10 to 575)	131 (91) (10 to 500)	83 (26) (10 to 202)	146 (91) (3 to 550)	91 (56) (10 to 400)	119 (60) (10 to 400)	148 (126) (10 to 550)	38 (18) (9 to 195)	57 (67) (1 to 581)	243 (141) (10 to 590)	114 (48) (40 to 315)	145 (78) (10 to 570)	234 (138) (11 to 500)	153 (142) (10 to 570)	112 (49) (11 to 400)
Unknown	452	35	3	116	27	2	40	1	44	0	4	47	360	0	2

（Continued）

Characteristic	Malaysia, N = 535[1]	Mexico, N = 1,016[1]	Nigeria, N = 590[1]	Philippines, N = 457[1]	Republic Of Sudan, N = 538[1]	Rwanda, N = 150[1]	Saudi Arabia, N = 631[1]	Singapore, N = 237[1]	South Africa, N = 198[1]	South Korea, N = 2,238[1]	Spain, N = 51[1]	Thailand, N = 723[1]	United Kingdom, N = 212[1]	United States, N = 213[1]	Vietnam, N = 419[1]
Age															
18-24	315 (59%)	389 (38%)	171 (29%)	225 (49%)	375 (70%)	10 (6·7%)	220 (35%)	80 (34%)	101 (51%)	161 (7·2%)	12 (24%)	152 (21%)	83 (39%)	19 (8·9%)	196 (47%)
25-29	23 (4·3%)	140 (14%)	255 (43%)	46 (10%)	85 (16%)	53 (35%)	121 (19%)	13 (5·5%)	19 (9·6%)	290 (13%)	2 (3·9%)	109 (15%)	27 (13%)	32 (15%)	34 (8·1%)
30-34	45 (8·4%)	95 (9·4%)	81 (14%)	54 (12%)	21 (3·9%)	44 (29%)	84 (13%)	26 (11%)	17 (8·6%)	227 (10%)	1 (2·0%)	136 (19%)	30 (14%)	32 (15%)	61 (15%)
35-39	25 (4·7%)	99 (9·7%)	38 (6·4%)	29 (6·3%)	9 (1·7%)	24 (16%)	73 (12%)	34 (14%)	20 (10%)	222 (9·9%)	5 (9·8%)	104 (14%)	25 (12%)	28 (13%)	77 (18%)
40-44	26 (4·9%)	101 (9·9%)	18 (3·1%)	20 (4·4%)	13 (2·4%)	12 (8·0%)	45 (7·1%)	29 (12%)	10 (5·1%)	273 (12%)	10 (20%)	93 (13%)	15 (7·1%)	21 (9·9%)	26 (6·2%)
45-49	45 (8·4%)	62 (6·1%)	13 (2·2%)	18 (3·9%)	12 (2·2%)	3 (2·0%)	44 (7·0%)	25 (11%)	13 (6·6%)	178 (8·0%)	10 (20%)	49 (6·8%)	8 (3·8%)	30 (14%)	16 (3·8%)
50-54	34 (6·4%)	47 (4·6%)	10 (1·7%)	27 (5·9%)	13 (2·4%)	1 (0·7%)	24 (3·8%)	12 (5·1%)	3 (1·5%)	288 (13%)	4 (7·8%)	32 (4·4%)	7 (3·3%)	14 (6·6%)	8 (1·9%)
55-59	10 (1·9%)	36 (3·5%)	3 (0·5%)	17 (3·7%)	4 (0·7%)	2 (1·3%)	10 (1·6%)	7 (3·0%)	9 (4·5%)	156 (7·0%)	4 (7·8%)	22 (3·0%)	7 (3·3%)	11 (5·2%)	1 (0·2%)
60-64	7 (1·3%)	31 (3·1%)	1 (0·2%)	6 (1·3%)	4 (0·7%)	1 (0·7%)	8 (1·3%)	6 (2·5%)	6 (3·0%)	321 (14%)	0 (0%)	10 (1·4%)	4 (1·9%)	11 (5·2%)	0 (0%)
>=65	5 (0·9%)	16 (1·6%)	0 (0%)	15 (3·3%)	2 (0·4%)	0 (0%)	2 (0·3%)	5 (2·1%)	0 (0%)	122 (5·5%)	3 (5·9%)	16 (2·2%)	6 (2·8%)	15 (7·0%)	0 (0%)
Gender															
Female	367 (69%)	661 (65%)	331 (56%)	324 (71%)	345 (64%)	41 (27%)	372 (59%)	169 (71%)	136 (69%)	1,128 (50%)	34 (67%)	512 (71%)	155 (73%)	191 (90%)	325 (78%)
Male	166 (31%)	347 (34%)	255 (43%)	124 (27%)	193 (36%)	106 (71%)	259 (41%)	66 (28%)	61 (31%)	1,103 (49%)	16 (31%)	192 (27%)	56 (26%)	20 (9·4%)	90 (21%)
Non-binary	2 (0·4%)	8 (0·8%)	4 (0·7%)	9 (2·0%)	0 (0%)	3 (2·0%)	0 (0%)	2 (0·8%)	1 (0·5%)	7 (0·3%)	1 (2·0%)	19 (2·6%)	1 (0·5%)	2 (0·9%)	4 (1·0%)
Marital															
Married/Cohabitation/Common-law	179 (33%)	309 (30%)	156 (26%)	124 (27%)	75 (14%)	69 (46%)	283 (45%)	109 (46%)	67 (34%)	1,331 (59%)	31 (61%)	168 (23%)	96 (45%)	127 (60%)	195 (47%)
Separated/Divorced/Widowed	7 (1·3%)	59 (5·8%)	7 (1·2%)	16 (3·5%)	9 (1·7%)	5 (3·3%)	20 (3·2%)	6 (2·5%)	7 (3·5%)	112 (5·0%)	2 (3·9%)	18 (2·5%)	9 (4·2%)	14 (6·6%)	7 (1·7%)
Single	349 (65%)	648 (64%)	427 (72%)	317 (69%)	454 (84%)	76 (51%)	328 (52%)	122 (51%)	124 (63%)	795 (36%)	18 (35%)	537 (74%)	107 (50%)	72 (34%)	217 (52%)
Education															
Primary or below	3 (0·6%)	12 (1·2%)	3 (0·5%)	3 (0·7%)	3 (0·6%)	0 (0%)	5 (0·8%)	1 (0·4%)	0 (0%)	3 (0·1%)	0 (0%)	3 (0·4%)	0 (0%)	1 (0·5%)	0 (0%)
Secondary	35 (6·5%)	206 (20%)	28 (4·7%)	58 (13%)	58 (11%)	25 (17%)	105 (17%)	2 (0·8%)	35 (18%)	16 (0·7%)	9 (18%)	25 (3·5%)	15 (7·1%)	2 (0·9%)	4 (1·0%)
College	89 (17%)	162 (16%)	166 (28%)	115 (25%)	22 (4·1%)	9 (6·0%)	21 (3·3%)	47 (20%)	23 (12%)	405 (18%)	12 (24%)	6 (0·8%)	49 (23%)	2 (0·9%)	7 (1·7%)

Associate degree	115 (21%)	3 (0·3%)	22 (3·7%)	9 (2·0%)	26 (4·8%)	8 (5·3%)	20 (3·2%)	1 (0·4%)	2 (1·0%)	344 (15%)	3 (5·9%)	8 (1·1%)	3 (1·4%)	2 (0·9%)	25 (6·0%)
Bachelor	265 (50%)	358 (35%)	299 (51%)	191 (42%)	392 (73%)	68 (45%)	389 (62%)	132 (56%)	105 (53%)	1,222 (55%)	5 (9·8%)	296 (41%)	86 (41%)	14 (6·6%)	296 (71%)
Graduate	27 (5·0%)	275 (27%)	72 (12%)	81 (18%)	37 (6·9%)	39 (26%)	90 (14%)	54 (23%)	33 (17%)	248 (11%)	22 (43%)	382 (53%)	58 (27%)	75 (35%)	87 (21%)
Unkown	1 (0·2%)	0 (0%)	0 (0%)	0 (0%)	0 (0%)	1 (0·7%)	1 (0·2%)	0 (0%)	0 (0%)	0 (0%)	0 (0%)	3 (0·4%)	1 (0·5%)	117 (55%)	0 (0%)
Employment	3 (0·6%)	12 (1·2%)	3 (0·5%)	3 (0·7%)	3 (0·6%)	0 (0%)	5 (0·8%)	1 (0·4%)	0 (0%)	3 (0·1%)	0 (0%)	3 (0·4%)	0 (0%)	1 (0·5%)	0 (0%)
Job seeking	16 (3·0%)	33 (3·2%)	65 (11%)	19 (4·2%)	42 (7·8%)	17 (11%)	79 (13%)	1 (0·4%)	8 (4·0%)	116 (5·2%)	2 (3·9%)	24 (3·3%)	5 (2·4%)	3 (1·4%)	8 (1·9%)
Laid off	1 (0·2%)	7 (0·7%)	4 (0·7%)	0 (0%)	1 (0·2%)	2 (1·3%)	5 (0·8%)	0 (0%)	1 (0·5%)	11 (0·5%)	0 (0%)	2 (0·3%)	2 (0·9%)	0 (0%)	0 (0%)
Not in workforce	17 (3·2%)	27 (2·7%)	8 (1·4%)	18 (3·9%)	20 (3·7%)	1 (0·7%)	60 (9·5%)	2 (0·8%)	2 (1·0%)	308 (14%)	0 (0%)	8 (1·1%)	3 (1·4%)	6 (2·8%)	0 (0%)
Retired	14 (2·6%)	19 (1·9%)	0 (0%)	14 (3·1%)	1 (0·2%)	0 (0%)	21 (3·3%)	5 (2·1%)	4 (2·0%)	69 (3·1%)	2 (3·9%)	20 (2·8%)	7 (3·3%)	9 (4·2%)	4 (1·0%)
Self-employed	19 (3·6%)	91 (9·0%)	28 (4·7%)	32 (7·0%)	31 (5·8%)	11 (7·3%)	8 (1·3%)	4 (1·7%)	19 (9·6%)	300 (13%)	9 (18%)	63 (8·7%)	9 (4·2%)	8 (3·8%)	39 (9·3%)
Student	309 (58%)	351 (35%)	200 (34%)	203 (44%)	352 (65%)	15 (10%)	211 (33%)	92 (39%)	95 (48%)	148 (6·6%)	12 (24%)	159 (22%)	103 (49%)	41 (19%)	163 (39%)
Working (>=40hrs/wk)	119 (22%)	299 (29%)	192 (33%)	132 (29%)	39 (7·2%)	78 (52%)	124 (20%)	117 (49%)	55 (28%)	988 (44%)	13 (25%)	325 (45%)	45 (21%)	106 (50%)	160 (38%)
Working (1-39hrs/wk)	40 (7·5%)	189 (19%)	93 (16%)	39 (8·5%)	52 (9·7%)	26 (17%)	123 (19%)	16 (6·8%)	14 (7·1%)	298 (13%)	13 (25%)	122 (17%)	38 (18%)	40 (19%)	45 (11%)
Height (m)	1·60 (0·08) (1·40 to 1·85)	1·64 (0·09) (1·23 to 2·10)	1·66 (0·14) (1·20 to 2·20)	1·60 (0·10) (1·15 to 1·96)	1·66 (0·11) (1·12 to 2·00)	1·68 (0·09) (1·40 to 1·93)	1·65 (0·10) (1·35 to 1·95)	1·64 (0·08) (1·48 to 1·85)	1·62 (0·15) (1·10 to 1·96)	1·67 (0·08) (1·40 to 1·90)	1·68 (0·08) (1·50 to 1·85)	1·63 (0·08) (1·43 to 1·85)	1·67 (0·11) (1·25 to 1·95)	1·65 (0·07) (1·45 to 1·80)	1·59 (0·07) (1·45 to 1·84)
Unknown	1	4	10	3	7	5	1	0	6	0	0	1	0	8	0
Weight(kg)	61 (13) (35 to 98)	69 (14) (40 to 120)	63 (11) (25 to 100)	62 (15) (21 to 112)	64 (14) (35 to 120)	68 (12) (32 to 97)	71 (19) (35 to 145)	64 (14) (39 to 103)	66 (18) (40 to 140)	65 (13) (32 to 137)	68 (13) (49 to 100)	62 (14) (37 to 108)	73 (18) (16 to 160)	73 (17) (40 to 134)	54 (9) (38 to 88)
Unknown	0	2	3	0	0	0	1	0	2	0	0	2	1	8	0
BMI	24·0 (4·6) (14·3 to 39·4)	25·6 (4·3) (13·8 to 39·4)	23·2 (5·3) (11·2 to 51·7)	24·3 (5·5) (7·5 to 47·4)	23·2 (4·7) (10·8 to 38·3)	24·0 (3·7) (12·9 to 32·9)	26·1 (5·7) (12·9 to 53·5)	23·5 (4·3) (15·2 to 36·9)	25·8 (7·7) (14·3 to 56·7)	23·2 (3·4) (13·3 to 45·7)	24·2 (3·6) (18·0 to 36·3)	23·1 (4·3) (14·1 to 38·9)	26·3 (6·4) (6·2 to 50·6)	26·8 (6·2) (16·9 to 50·2)	21·2 (2·7) (14·7 to 32·9)
Unknown	1	6	11	3	7	5	1	0	7	0	0	2	1	11	0
BMI classification															
Severely underweight	14 (2·6%)	4 (0·4%)	30 (5·2%)	11 (2·4%)	26 (4·9%)	3 (2·1%)	7 (1·1%)	2 (0·8%)	4 (2·1%)	11 (0·5%)	0 (0%)	13 (1·8%)	3 (1·4%)	0 (0%)	7 (1·7%)

Underweight	37 (6·9%)	28 (2·8%)	49 (8·5%)	35 (7·7%)	50 (9·4%)	1 (0·7%)	33 (5·2%)	21 (8·9%)	19 (9·9%)	108 (4·8%)	2 (3·9%)	60 (8·3%)	7 (3·3%)	2 (1·0%)	53 (13%)
Normal weight	199 (37%)	440 (44%)	343 (59%)	175 (39%)	279 (53%)	90 (62%)	155 (25%)	99 (42%)	80 (42%)	1,032 (46%)	32 (63%)	338 (47%)	90 (43%)	92 (46%)	264 (63%)
Overweight	85 (16%)	378 (37%)	101 (17%)	63 (14%)	124 (23%)	42 (29%)	95 (15%)	35 (15%)	45 (24%)	485 (22%)	14 (27%)	116 (16%)	72 (34%)	61 (30%)	58 (14%)
Obesity	199 (37%)	160 (16%)	56 (9·7%)	170 (37%)	52 (9·8%)	9 (6·2%)	340 (54%)	80 (34%)	43 (23%)	602 (27%)	3 (5·9%)	194 (27%)	39 (18%)	47 (23%)	37 (8·8%)
Unknown	1	6	11	3	7	5	1	0	7	0	0	2	1	11	0
Pregnant															
Not applicable	166 (31%)	347 (34%)	255 (43%)	124 (27%)	193 (36%)	106 (71%)	259 (41%)	66 (28%)	61 (31%)	1,110 (50%)	16 (31%)	192 (27%)	56 (26%)	22 (10%)	90 (21%)
No	363 (68%)	665 (65%)	320 (54%)	330 (72%)	342 (64%)	40 (27%)	356 (56%)	170 (72%)	136 (69%)	1,119 (50%)	35 (69%)	527 (73%)	156 (74%)	187 (88%)	317 (76%)
Yes	6 (1·1%)	4 (0·4%)	15 (2·5%)	3 (0·7%)	3 (0·6%)	4 (2·7%)	16 (2·5%)	1 (0·4%)	1 (0·5%)	9 (0·4%)	0 (0%)	4 (0·6%)	0 (0%)	4 (1·9%)	12 (2·9%)
Gestational week	25 (12) (12 to 35)	24 (NA) (24 to 24)	27 (8) (17 to 34)	NA (NA) (Inf to -Inf)	7 (1) (6 to 8)	6 (3) (4 to 9)	15 (11) (5 to 30)	10 (NA) (10 to 10)	NA (NA) (Inf to -Inf)	9 (2) (5 to 10)	NA (NA) (Inf to -Inf)	18 (15) (6 to 36)	NA (NA) (Inf to -Inf)	29 (4) (25 to 34)	31 (8) (15 to 36)
Unknown	532	1,015	586	457	536	147	626	236	198	2,229	51	719	212	209	413
Regular medical follow-up before COVID-19															
NA	0 (0%)	0 (0%)	0 (0%)	0 (0%)	0 (0%)	0 (0%)	0 (0%)	0 (0%)	0 (0%)	0 (0%)	0 (0%)	0 (0%)	0 (0%)	2 (0·9%)	0 (0%)
No	436 (81%)	702 (69%)	469 (79%)	344 (75%)	450 (84%)	139 (93%)	448 (71%)	187 (79%)	151 (76%)	1,372 (61%)	35 (69%)	502 (69%)	154 (73%)	88 (41%)	62 (15%)
Yes	99 (19%)	314 (31%)	121 (21%)	113 (25%)	88 (16%)	11 (7·3%)	183 (29%)	50 (21%)	47 (24%)	866 (39%)	16 (31%)	221 (31%)	58 (27%)	123 (58%)	357 (85%)
Practising healthcare professional															
NA	0 (0%)	0 (0%)	0 (0%)	0 (0%)	0 (0%)	0 (0%)	0 (0%)	0 (0%)	0 (0%)	0 (0%)	0 (0%)	0 (0%)	0 (0%)	0 (0%)	0 (0%)
No	290 (54%)	425 (42%)	276 (47%)	267 (58%)	357 (66%)	71 (47%)	466 (74%)	124 (52%)	133 (67%)	2,150 (96%)	42 (82%)	634 (88%)	162 (76%)	81 (38%)	229 (55%)
Yes	245 (46%)	591 (58%)	314 (53%)	190 (42%)	181 (34%)	79 (53%)	165 (26%)	113 (48%)	65 (33%)	88 (3·9%)	9 (18%)	89 (12%)	50 (24%)	132 (62%)	190 (45%)
Number of children less than 18 years old															
No	398 (74%)	802 (79%)	468 (79%)	352 (77%)	484 (90%)	87 (58%)	390 (62%)	164 (69%)	163 (82%)	1,634 (73%)	33 (65%)	631 (87%)	163 (77%)	147 (69%)	242 (58%)
Yes	137 (26%)	214 (21%)	122 (21%)	105 (23%)	54 (10%)	63 (42%)	241 (38%)	73 (31%)	35 (18%)	604 (27%)	18 (35%)	92 (13%)	49 (23%)	66 (31%)	177 (42%)

Number of children aged above 18	0·58 (1·17) (0·00 to 8·00)	0·34 (0·84) (0·00 to 11·00)	0·48 (1·21) (0·00 to 10·00)	0·36 (0·79) (0·00 to 4·00)	0·26 (0·90) (0·00 to 6·00)	0·83 (1·20) (0·00 to 5·00)	0·99 (1·55) (0·00 to 7·00)	0·61 (1·02) (0·00 to 4·00)	0·30 (0·71) (0·00 to 3·00)	0·42 (0·76) (0·00 to 5·00)	0·63 (0·94) (0·00 to 3·00)	0·18 (0·52) (0·00 to 4·00)	0·41 (0·90) (0·00 to 6·00)	0·49 (0·83) (0·00 to 4·00)	0·76 (0·96) (0·00 to 4·00)
Unknown	0	11	3	2	3	0	17	0	0	0	0	2	1	1	0
Number of people lived with	5·07 (2·17) (1·00 to 16·00)	3·86 (1·83) (1·00 to 14·00)	4·26 (2·61) (1·00 to 23·00)	4·97 (2·25) (1·00 to 14·00)	6·59 (2·60) (1·00 to 18·00)	3·83 (2·21) (1·00 to 10·00)	6·25 (2·76) (1·00 to 21·00)	4·21 (1·47) (1·00 to 10·00)	4·33 (2·16) (1·00 to 12·00)	3·05 (1·15) (1·00 to 7·00)	3·35 (1·38) (1·00 to 6·00)	3·35 (1·91) (1·00 to 18·00)	3·14 (1·49) (1·00 to 8·00)	2·67 (1·31) (1·00 to 8·00)	4·31 (2·03) (1·00 to 12·00)
Unknown	2	2	4	5	35	0	32	0	1	0	0	1	0	10	6
Perceived social rank	3·49 (0·81) (1·00 to 5·00)	3·01 (0·64) (1·00 to 5·00)	2·99 (0·90) (1·00 to 5·00)	3·27 (0·70) (1·00 to 5·00)	3·17 (1·03) (1·00 to 5·00)	2·83 (0·75) (1·00 to 5·00)	3·46 (0·97) (1·00 to 5·00)	3·32 (0·71) (1·00 to 5·00)	3·19 (1·01) (1·00 to 5·00)	2·95 (0·70) (1·00 to 5·00)	3·00 (0·66) (1·00 to 5·00)	3·84 (0·87) (1·00 to 5·00)	3·34 (0·77) (1·00 to 5·00)	3·77 (0·76) (1·00 to 5·00)	3·23 (0·72) (1·00 to 5·00)
Unknown	0	0	0	0	0	0	0	0	0	0	0	0	0	2	0
House size	136 (127) (1 to 594)	125 (99) (10 to 500)	68 (80) (1 to 555)	120 (107) (1 to 561)	243 (145) (10 to 500)	98 (45) (12 to 249)	247 (155) (10 to 555)	115 (71) (1 to 500)	146 (124) (1 to 550)	31 (13) (10 to 100)	110 (62) (25 to 300)	134 (119) (10 to 540)	98 (89) (1 to 500)	195 (109) (20 to 560)	168 (141) (10 to 650)
Unknown	17	85	19	20	226	1	296	3	8	1	0	56	22	42	19

[1]n (%); Mean (SD) (Range)

eTable 3. Weighted demographic summary by country.

Characteristic	Australia, N = 639[1]	Brazil, N = 553[1]	Burundi, N = 369[1]	Canada, N = 368[1]	Chile, N = 342[1]	Egypt, N = 461[1]	Guatemala, N = 229[1]	Hong Kong, N = 2,127[1]	India, N = 529[1]	Indonesia, N = 405[1]	Italy, N = 203[1]	Lebanon, N = 440[1]	Libya, N = 612[1]	Macau, N = 233[1]	Mainland China, N = 667[1]
Age															
18-24	59 (9·2%)	75 (13%)	80 (22%)	40 (11%)	34 (10·0%)	71 (15%)	41 (18%)	154 (7·2%)	80 (15%)	69 (17%)	12 (6·0%)	59 (13%)	91 (15%)	19 (8·3%)	101 (15%)
25-29	64 (10·0%)	74 (13%)	70 (19%)	30 (8·1%)	39 (11%)	71 (15%)	37 (16%)	176 (8·3%)	73 (14%)	69 (17%)	13 (6·4%)	51 (11%)	95 (15%)	22 (9·5%)	83 (12%)
30-34	63 (9·9%)	68 (12%)	54 (15%)	30 (8·3%)	38 (11%)	63 (14%)	31 (14%)	199 (9·4%)	66 (12%)	67 (17%)	14 (6·7%)	43 (9·9%)	94 (15%)	33 (14%)	76 (11%)
35-39	60 (9·3%)	60 (11%)	38 (10%)	30 (8·1%)	34 (9·9%)	56 (12%)	26 (11%)	197 (9·3%)	60 (11%)	66 (16%)	15 (7·3%)	40 (9·1%)	91 (15%)	29 (12%)	90 (13%)
40-44	53 (8·3%)	56 (10%)	30 (8·2%)	30 (8·0%)	32 (9·3%)	45 (9·7%)	21 (9·4%)	197 (9·2%)	54 (10%)	31 (7·7%)	17 (8·5%)	37 (8·3%)	77 (13%)	22 (9·6%)	100 (15%)
45-49	56 (8·8%)	51 (9·3%)	26 (6·9%)	31 (8·4%)	30 (8·8%)	38 (8·2%)	18 (7·7%)	196 (9·2%)	47 (9·0%)	57 (14%)	20 (9·7%)	36 (8·3%)	60 (9·8%)	22 (9·6%)	94 (14%)
50-54	51 (8·0%)	44 (7·9%)	22 (5·9%)	35 (9·5%)	29 (8·5%)	35 (7·5%)	14 (6·1%)	222 (10%)	39 (7·5%)	25 (6·2%)	20 (9·9%)	39 (8·9%)	42 (6·9%)	21 (9·0%)	58 (8·7%)
55-59	52 (8·1%)	36 (6·5%)	19 (5·0%)	34 (9·3%)	27 (7·9%)	28 (6·1%)	11 (4·9%)	215 (10%)	32 (6·0%)	21 (5·2%)	19 (9·3%)	34 (7·8%)	29 (4·7%)	24 (10%)	66 (10·0%)
60-64	46 (7·3%)	28 (5·1%)	12 (3·2%)	30 (8·1%)	23 (6·8%)	23 (5·0%)	9 (3·9%)	171 (8·0%)	25 (4·8%)	0 (0%)	16 (7·9%)	29 (6·5%)	9 (1·5%)	20 (8·8%)	0 (0%)
>=65	135 (21%)	61 (11%)	18 (4·9%)	78 (21%)	56 (16%)	32 (6·9%)	21 (9·4%)	401 (19%)	53 (10·0%)	0 (0%)	57 (28%)	72 (16%)	24 (3·9%)	19 (8·3%)	0 (0%)
Gender															
Female	324 (51%)	287 (52%)	178 (48%)	186 (50%)	173 (51%)	224 (49%)	117 (51%)	1,171 (55%)	260 (49%)	184 (45%)	105 (52%)	207 (47%)	287 (47%)	133 (57%)	327 (49%)
Male	312 (49%)	266 (48%)	172 (47%)	174 (47%)	164 (48%)	237 (51%)	107 (47%)	955 (45%)	269 (51%)	222 (55%)	98 (48%)	233 (53%)	326 (53%)	99 (43%)	334 (50%)
Non-binary	3 (0·5%)	0 (<0·1%)	18 (5·0%)	9 (2·3%)	5 (1·4%)	0 (0%)	4 (1·9%)	1 (<0·1%)	0 (0%)	0 (0%)	0 (0%)	0 (0%)	0 (0%)	0 (0%)	6 (0·9%)
Marital															
Single	188 (29%)	197 (36%)	155 (42%)	89 (24%)	139 (41%)	130 (28%)	116 (50%)	559 (26%)	147 (28%)	158 (39%)	35 (17%)	125 (28%)	268 (44%)	70 (30%)	200 (30%)
Married/Cohabitation/Common-law	350 (55%)	306 (55%)	190 (51%)	239 (65%)	144 (42%)	306 (66%)	109 (48%)	1,411 (66%)	333 (63%)	248 (61%)	142 (70%)	269 (61%)	324 (53%)	154 (66%)	411 (62%)
Separated/Divorced/Widowed	100 (16%)	50 (9·1%)	24 (6·5%)	39 (11%)	59 (17%)	25 (5·4%)	5 (2·0%)	156 (7·3%)	49 (9·2%)	0 (0%)	26 (13%)	46 (10%)	20 (3·3%)	9 (3·9%)	56 (8·3%)
Unknown	1	0	0	0	0	0	0	0	0	0	0	0	0	0	0
Education															
Primary or below	0 (0%)	2 (0·3%)	133 (36%)	0 (0·1%)	5 (1·4%)	1 (0·1%)	0 (0%)	371 (17%)	74 (14%)	0 (0%)	4 (2·2%)	37 (8·4%)	6 (1·0%)	0 (0%)	7 (1·1%)
Secondary	163 (27%)	57 (10%)	178 (48%)	51 (14%)	38 (11%)	23 (5·0%)	30 (13%)	1,072 (50%)	63 (12%)	0 (0%)	11 (5·3%)	49 (11%)	61 (10%)	12 (5·1%)	134 (20%)

Associate degree	119 (20%)	9 (1·7%)	14 (3·7%)	10 (2·8%)	4 (1·2%)	16 (3·4%)	2 (0·7%)	158 (7·4%)	44 (8·4%)	73 (18%)	66 (32%)	40 (9·2%)	51 (8·4%)	114 (49%)	17 (2·6%)
College	179 (30%)	53 (9·5%)	13 (3·6%)	104 (28%)	128 (37%)	45 (9·8%)	62 (27%)	143 (6·7%)	65 (13%)	27 (6·6%)	13 (6·2%)	43 (9·7%)	70 (11%)	9 (3·8%)	176 (27%)
Bachelor	79 (13%)	144 (26%)	24 (6·6%)	96 (26%)	47 (14%)	201 (44%)	44 (19%)	342 (16%)	102 (20%)	195 (48%)	17 (8·1%)	127 (29%)	284 (46%)	78 (34%)	158 (24%)
Graduate	55 (9·2%)	288 (52%)	6 (1·7%)	107 (29%)	119 (35%)	175 (38%)	92 (40%)	41 (1·9%)	173 (33%)	110 (27%)	93 (46%)	144 (33%)	139 (23%)	19 (8·2%)	163 (25%)
Unknown	44	0	0	0	0	0	0	0	7	0	0	0	0	0	12
Employment															
Job seeking	84 (13%)	9 (1·7%)	18 (5·0%)	12 (3·3%)	24 (7·1%)	17 (3·8%)	6 (2·6%)	60 (2·8%)	27 (5·0%)	10 (2·4%)	6 (2·9%)	38 (8·7%)	35 (5·7%)	1 (0·5%)	52 (7·8%)
Laid off	44 (6·9%)	1 (0·2%)	13 (3·6%)	8 (2·0%)	0 (0%)	0 (0%)	3 (1·2%)	36 (1·7%)	0 (<0·1%)	0 (0%)	0 (0%)	7 (1·5%)	1 (0·2%)	0 (0%)	22 (3·2%)
Not in workforce	44 (6·9%)	8 (1·4%)	3 (0·9%)	34 (9·2%)	14 (4·0%)	34 (7·3%)	0 (0%)	231 (11%)	95 (18%)	3 (0·7%)	7 (3·6%)	63 (14%)	34 (5·5%)	3 (1·2%)	18 (2·8%)
Retired	58 (9·0%)	63 (11%)	3 (0·8%)	91 (25%)	52 (15%)	48 (10%)	12 (5·1%)	437 (21%)	22 (4·3%)	0 (0%)	62 (30%)	5 (1·2%)	24 (3·9%)	23 (9·7%)	35 (5·3%)
Self-employed	7 (1·1%)	63 (11%)	179 (48%)	20 (5·4%)	26 (7·7%)	68 (15%)	34 (15%)	37 (1·7%)	78 (15%)	73 (18%)	21 (10%)	108 (25%)	58 (9·4%)	14 (6·2%)	40 (6·1%)
Student	39 (6·1%)	83 (15%)	82 (22%)	25 (6·9%)	50 (15%)	64 (14%)	57 (25%)	127 (6·0%)	92 (17%)	90 (22%)	12 (6·1%)	49 (11%)	114 (19%)	16 (6·7%)	102 (15%)
Working (>=40hrs/wk)	66 (10%)	239 (43%)	22 (6·1%)	82 (22%)	143 (42%)	129 (28%)	78 (34%)	885 (42%)	158 (30%)	112 (28%)	46 (22%)	77 (18%)	111 (18%)	89 (38%)	280 (42%)
Working (1-39hrs/wk)	297 (46%)	87 (16%)	47 (13%)	97 (26%)	32 (9·4%)	100 (22%)	40 (17%)	313 (15%)	57 (11%)	117 (29%)	49 (24%)	92 (21%)	236 (39%)	87 (37%)	118 (18%)
Height (m)	1·70 (0·08) (1·50 to 1·96)	1·69 (0·11) (1·16 to 1·94)	1·63 (0·06) (1·52 to 1·90)	1·70 (0·12) (1·28 to 2·11)	1·67 (0·09) (1·40 to 1·90)	1·68 (0·10) (1·30 to 1·97)	1·66 (0·10) (1·44 to 1·98)	1·64 (0·08) (1·37 to 1·98)	1·63 (0·10) (1·43 to 1·96)	1·62 (0·07) (1·45 to 1·84)	1·70 (0·09) (1·50 to 1·94)	1·68 (0·09) (1·47 to 1·98)	1·66 (0·14) (1·20 to 2·00)	1·65 (0·08) (1·49 to 1·98)	1·67 (0·08) (1·30 to 1·86)
Unknown	14	0	1	5	0	0	1	1	4	0	0	1	3	0	5
Weight(kg)	74 (14) (27 to 130)	74 (16) (37 to 120)	60 (7) (45 to 89)	79 (19) (31 to 127)	75 (13) (41 to 113)	80 (17) (40 to 160)	69 (14) (39 to 110)	62 (10) (39 to 117)	67 (12) (34 to 104)	64 (13) (33 to 103)	72 (16) (45 to 122)	75 (15) (40 to 140)	76 (18) (36 to 150)	63 (12) (30 to 110)	66 (14) (35 to 135)
Unknown	8	0	0	26	0	0	1	0	5	0	0	0	0	0	5
BMI	25·6 (4·2) (11·7 to 45·8)	26·0 (4·5) (15·6 to 42·0)	22·4 (2·2) (15·5 to 34·0)	27·0 (5·2) (13·5 to 43·8)	26·7 (3·8) (17·6 to 44·4)	28·4 (5·3) (15·4 to 62·4)	24·8 (3·9) (13·9 to 37·1)	23·1 (3·0) (13·4 to 38·2)	25·1 (3·7) (14·5 to 36·1)	24·1 (4·5) (14·1 to 40·3)	24·9 (4·4) (17·3 to 38·9)	26·6 (4·3) (13·8 to 48·4)	27·9 (7·5) (12·9 to 52·1)	22·9 (3·5) (11·0 to 37·5)	23·5 (4·2) (12·1 to 48·4)
Unknown	16	0	1	28	0	0	2	1	5	0	0	1	3	0	5
BMI classification															
Severely underweight	6 (1·0%)	2 (0·3%)	4 (1·0%)	1 (0·4%)	0 (0%)	1 (0·2%)	5 (2·0%)	7 (0·3%)	3 (0·5%)	3 (0·7%)	0 (0%)	0 (<0·1%)	5 (0·7%)	5 (2·3%)	4 (0·6%)
Underweight	16 (2·6%)	12 (2·2%)	3 (0·9%)	5 (1·4%)	2 (0·5%)	6 (1·2%)	2 (0·8%)	101 (4·7%)	12 (2·4%)	36 (8·9%)	4 (1·8%)	10 (2·2%)	12 (2·0%)	17 (7·5%)	41 (6·2%)
Normal weight	260 (42%)	238 (43%)	336 (91%)	120 (35%)	105 (31%)	51 (11%)	115 (51%)	868 (41%)	134 (26%)	144 (35%)	114 (56%)	76 (17%)	221 (36%)	86 (37%)	264 (40%)

Overweight	248 (40%)	194 (35%)	18 (4·9%)	128 (38%)	171 (50%)	68 (15%)	84 (37%)	634 (30%)	106 (20%)	59 (15%)	63 (31%)	77 (18%)	206 (34%)	62 (27%)	172 (26%)
Obesity	93 (15%)	108 (19%)	7 (1·8%)	85 (25%)	65 (19%)	336 (73%)	21 (9·2%)	516 (24%)	268 (51%)	164 (40%)	23 (11%)	276 (63%)	165 (27%)	62 (27%)	182 (27%)
Unknown	16	0	1	28	0	0	2	1	5	0	0	1	3	0	5
Pregnant															
Not applicable	313 (49%)	266 (48%)	172 (47%)	174 (47%)	164 (48%)	254 (55%)	107 (47%)	955 (45%)	269 (51%)	222 (55%)	98 (48%)	238 (54%)	326 (53%)	99 (43%)	335 (50%)
No	321 (50%)	283 (51%)	164 (44%)	185 (50%)	176 (52%)	207 (45%)	119 (52%)	1,165 (55%)	259 (49%)	176 (43%)	104 (51%)	200 (45%)	273 (45%)	106 (45%)	257 (39%)
Yes	5 (0·8%)	4 (0·8%)	33 (9·0%)	9 (2·5%)	1 (0·4%)	0 (0%)	3 (1·1%)	7 (0·3%)	1 (0·1%)	8 (2·0%)	0 (0·2%)	3 (0·6%)	14 (2·2%)	28 (12%)	75 (11%)
Gestational week	20 (12) (6 to 40)	7 (0) (6 to 7)	5 (1) (3 to 7)	25 (11) (4 to 39)	30 (0) (30 to 30)	0 (0) (Inf to -Inf)	0 (0) (Inf to -Inf)	25 (9) (15 to 40)	0 (0) (Inf to -Inf)	11 (4) (8 to 32)	12 (0) (12 to 12)	17 (11) (6 to 30)	24 (11) (9 to 36)	38 (7) (5 to 40)	35 (7) (4 to 41)
Unknown	634	552	354	359	341	461	229	2,120	529	397	203	437	607	205	643
Regular medical follow-up before COVID-19															
No	435 (68%)	374 (68%)	340 (92%)	166 (45%)	199 (58%)	316 (69%)	157 (69%)	1,363 (64%)	320 (61%)	261 (65%)	53 (26%)	223 (51%)	427 (70%)	156 (67%)	570 (85%)
Yes	204 (32%)	179 (32%)	29 (7·8%)	202 (55%)	143 (42%)	145 (31%)	72 (31%)	764 (36%)	209 (39%)	144 (35%)	150 (74%)	217 (49%)	185 (30%)	77 (33%)	97 (15%)
Unknown	1	0	0	0	0	0	0	0	0	0	0	0	0	0	0
Practising healthcare professional															
No	557 (87%)	365 (66%)	354 (96%)	345 (94%)	303 (89%)	249 (54%)	166 (73%)	2,065 (97%)	389 (73%)	225 (55%)	189 (93%)	311 (71%)	420 (69%)	117 (50%)	619 (93%)
Yes	82 (13%)	188 (34%)	15 (4·2%)	23 (6·3%)	39 (11%)	212 (46%)	63 (27%)	62 (2·9%)	140 (27%)	181 (45%)	14 (7·0%)	129 (29%)	192 (31%)	116 (50%)	48 (7·2%)
Unknown	0	0	0	0	0	0	0	0	0	0	0	0	0	0	0
Number of children less than 18 years old															
No	526 (82%)	397 (72%)	193 (52%)	270 (73%)	240 (70%)	233 (50%)	172 (75%)	1,591 (75%)	325 (61%)	255 (63%)	152 (75%)	284 (65%)	372 (61%)	125 (54%)	360 (54%)
Yes	113 (18%)	156 (28%)	176 (48%)	98 (27%)	102 (30%)	228 (50%)	57 (25%)	536 (25%)	204 (39%)	151 (37%)	51 (25%)	156 (35%)	240 (39%)	107 (46%)	307 (46%)
Number of children aged above 18	0·30 (0·70) (0·00 to 3·00)	0·43 (0·86) (0·00 to 8·00)	1·00 (1·21) (0·00 to 6·00)	0·49 (0·95) (0·00 to 5·00)	0·45 (0·83) (0·00 to 5·00)	1·03 (1·25) (0·00 to 5·00)	0·41 (0·82) (0·00 to 5·00)	0·34 (0·64) (0·00 to 4·00)	0·59 (0·86) (0·00 to 5·00)	0·44 (0·62) (0·00 to 2·00)	0·40 (0·76) (0·00 to 3·00)	0·84 (1·26) (0·00 to 5·00)	1·13 (1·66) (0·00 to 6·00)	0·79 (1·00) (0·00 to 7·00)	0·57 (0·73) (0·00 to 6·00)
Unknown	0	10	0	1	4	10	3	0	1	0	0	0	12	0	10

Number of people lived with	2·74 (1·19) (1·00 to 6·00)	2·77 (1·28) (1·00 to 7·00)	4·11 (1·46) (1·00 to 8·00)	2·46 (1·31) (1·00 to 7·00)	3·01 (1·41) (1·00 to 8·00)	4·50 (1·85) (1·00 to 17·00)	4·01 (2·09) (1·00 to 12·00)	3·27 (1·13) (1·00 to 9·00)	3·76 (1·69) (1·00 to 20·00)	4·38 (1·31) (1·00 to 9·00)	2·51 (1·18) (1·00 to 6·00)	4·25 (1·45) (1·00 to 11·00)	5·90 (2·97) (1·00 to 26·00)	3·87 (1·60) (1·00 to 12·00)	3·22 (1·17) (1·00 to 10·00)
Unknown	41	0	4	1	0	3	0	0	2	0	1	0	16	0	49
Perceived social rank	3·15 (0·93) (1·00 to 5·00)	3·51 (0·83) (1·00 to 5·00)	1·69 (0·76) (1·00 to 4·00)	3·43 (0·97) (1·00 to 5·00)	3·19 (0·85) (1·00 to 5·00)	3·41 (0·93) (1·00 to 5·00)	3·26 (0·73) (1·00 to 5·00)	2·69 (0·78) (1·00 to 5·00)	2·97 (1·16) (1·00 to 5·00)	3·45 (0·83) (1·00 to 5·00)	3·57 (0·72) (2·00 to 5·00)	2·99 (0·78) (1·00 to 5·00)	3·25 (1·10) (1·00 to 5·00)	3·41 (0·70) (1·00 to 5·00)	2·81 (0·79) (1·00 to 5·00)
Unknown	1	0	0	0	0	0	0	0	0	11	0	0	0	0	1
House size	175 (141) (10 to 575)	137 (89) (10 to 500)	85 (31) (10 to 202)	137 (84) (3 to 550)	92 (51) (10 to 400)	119 (56) (10 to 400)	163 (118) (10 to 550)	39 (19) (9 to 195)	53 (61) (1 to 581)	194 (117) (10 to 590)	115 (47) (40 to 315)	135 (66) (10 to 570)	244 (144) (11 to 500)	140 (117) (10 to 570)	105 (46) (11 to 400)
Unknown	431	29	2	113	31	2	23	1	30	0	13	21	281	0	5

(Continued)

Characteristic	Malaysia, N = 535[1]	Mexico, N = 1,016[1]	Nigeria, N = 580[1]	Philippines, N = 457[1]	Republic Of Sudan, N = 538[1]	Rwanda, N = 136[1]	Saudi Arabia, N = 609[1]	Singapore, N = 237[1]	South Africa, N = 192[1]	South Korea, N = 2,238[1]	Spain, N = 45[1]	Thailand, N = 723[1]	United Kingdom, N = 212[1]	United States, N = 184[1]	Vietnam, N = 401[1]
Age															
18-24	75 (14%)	127 (13%)	127 (22%)	74 (16%)	108 (20%)	29 (21%)	70 (12%)	24 (10%)	28 (15%)	185 (8·3%)	3 (7·6%)	70 (9·7%)	18 (8·3%)	19 (10%)	51 (13%)
25-29	78 (15%)	122 (12%)	116 (20%)	65 (14%)	90 (17%)	26 (19%)	88 (14%)	20 (8·6%)	31 (16%)	185 (8·3%)	2 (4·1%)	76 (11%)	19 (9·0%)	20 (11%)	64 (16%)
30-34	69 (13%)	115 (11%)	90 (15%)	57 (13%)	74 (14%)	24 (18%)	89 (15%)	21 (8·9%)	33 (17%)	179 (8·0%)	2 (4·5%)	81 (11%)	19 (8·8%)	19 (10%)	64 (16%)
35-39	62 (12%)	110 (11%)	69 (12%)	53 (11%)	69 (13%)	21 (15%)	100 (16%)	21 (9·0%)	28 (14%)	217 (9·7%)	2 (5·4%)	84 (12%)	18 (8·5%)	19 (10%)	59 (15%)
40-44	48 (9·0%)	104 (10%)	61 (11%)	46 (10·0%)	53 (9·9%)	15 (11%)	89 (15%)	21 (9·0%)	22 (11%)	210 (9·4%)	6 (13%)	85 (12%)	17 (7·9%)	17 (9·3%)	51 (13%)
45-49	43 (8·0%)	97 (9·5%)	44 (7·5%)	41 (9·0%)	46 (8·6%)	5 (3·6%)	66 (11%)	22 (9·4%)	18 (9·6%)	242 (11%)	6 (13%)	79 (11%)	19 (9·0%)	18 (9·8%)	48 (12%)
50-54	39 (7·4%)	86 (8·5%)	40 (6·9%)	35 (7·6%)	32 (6·0%)	4 (3·0%)	46 (7·6%)	21 (8·9%)	15 (7·8%)	219 (9·8%)	5 (12%)	69 (9·5%)	20 (9·2%)	18 (9·8%)	43 (11%)
55-59	35 (6·6%)	70 (6·8%)	20 (3·4%)	28 (6·2%)	25 (4·7%)	8 (6·0%)	32 (5·2%)	22 (9·2%)	13 (6·7%)	230 (10%)	5 (11%)	53 (7·4%)	18 (8·4%)	19 (10%)	21 (5·2%)
60-64	29 (5·4%)	59 (5·8%)	13 (2·2%)	22 (4·7%)	17 (3·2%)	4 (2·7%)	13 (2·2%)	20 (8·5%)	4 (2·3%)	180 (8·0%)	0 (0%)	41 (5·7%)	15 (7·2%)	9 (5·0%)	0 (0%)
>=65	55 (10%)	126 (12%)	0 (0%)	37 (8·2%)	23 (4·3%)	0 (0%)	16 (2·6%)	44 (18%)	0 (0%)	391 (17%)	13 (30%)	86 (12%)	51 (24%)	25 (14%)	0 (0%)
Gender															
Female	260 (49%)	529 (52%)	287 (49%)	223 (49%)	262 (49%)	65 (48%)	225 (37%)	122 (52%)	95 (49%)	1,121 (50%)	26 (58%)	366 (51%)	109 (51%)	109 (59%)	208 (52%)
Male	274 (51%)	479 (47%)	290 (50%)	225 (49%)	276 (51%)	66 (49%)	384 (63%)	104 (44%)	97 (50%)	1,110 (50%)	18 (41%)	344 (48%)	103 (49%)	72 (39%)	190 (48%)
Non-binary	1 (0·2%)	8 (0·8%)	3 (0·4%)	9 (2·0%)	0 (0%)	5 (3·5%)	0 (0%)	11 (4·7%)	0 (<0·1%)	6 (0·3%)	0 (1·1%)	14 (1·9%)	0 (0·2%)	3 (1·8%)	2 (0·5%)
Marital															
Single	159 (30%)	433 (43%)	281 (48%)	208 (46%)	254 (47%)	59 (43%)	174 (29%)	93 (39%)	77 (40%)	686 (31%)	9 (19%)	424 (59%)	45 (21%)	64 (35%)	105 (26%)
Married/Cohabitation/Common-law	353 (66%)	432 (43%)	278 (48%)	220 (48%)	244 (45%)	70 (51%)	411 (67%)	137 (58%)	106 (55%)	1,427 (64%)	32 (71%)	256 (35%)	146 (69%)	108 (59%)	284 (71%)
Separated/Divorced/Widowed	24 (4·5%)	151 (15%)	20 (3·5%)	29 (6·3%)	40 (7·5%)	8 (5·7%)	24 (3·9%)	7 (3·0%)	9 (4·7%)	125 (5·6%)	4 (9·6%)	43 (6·0%)	20 (9·6%)	12 (6·4%)	12 (2·9%)
Unknown	0	0	0	0	0	0	0	0	0	0	0	0	0	0	0
Education															
Primary or below	24 (4·5%)	14 (1·4%)	2 (0·4%)	2 (0·4%)	24 (4·4%)	0 (0%)	4 (0·7%)	1 (0·3%)	0 (0%)	2 (<0·1%)	0 (0%)	14 (1·9%)	0 (0%)	3 (3·0%)	0 (0%)
Secondary	57 (11%)	95 (9·4%)	23 (3·9%)	23 (5·0%)	52 (9·7%)	31 (23%)	86 (14%)	2 (0·9%)	27 (14%)	30 (1·3%)	3 (6·4%)	17 (2·4%)	12 (5·7%)	1 (1·2%)	9 (2·3%)

Associate degree	143 (27%)	3 (0·3%)	16 (2·8%)	10 (2·3%)	25 (4·6%)	8 (6·0%)	13 (2·1%)	1 (0·6%)	2 (1·3%)	316 (14%)	1 (1·1%)	22 (3·0%)	2 (1·2%)	3 (2·8%)	36 (9·0%)
College	112 (21%)	122 (12%)	143 (25%)	74 (16%)	26 (4·8%)	10 (7·2%)	26 (4·3%)	37 (16%)	22 (11%)	464 (21%)	16 (37%)	6 (0·9%)	52 (25%)	10 (10%)	22 (5·5%)
Bachelor	153 (29%)	320 (32%)	249 (43%)	216 (47%)	284 (53%)	47 (34%)	324 (53%)	132 (56%)	89 (46%)	1,165 (52%)	4 (7·9%)	199 (28%)	62 (29%)	22 (23%)	188 (47%)
Graduate	44 (8·3%)	461 (45%)	147 (25%)	133 (29%)	128 (24%)	40 (30%)	154 (25%)	63 (27%)	52 (27%)	261 (12%)	21 (48%)	462 (64%)	82 (39%)	57 (60%)	145 (36%)
Unknown	2	0	0	0	0	0	2	0	0	0	0	3	2	89	0
Employment															
Job seeking	15 (2·9%)	28 (2·7%)	49 (8·5%)	19 (4·2%)	34 (6·3%)	13 (9·2%)	50 (8·2%)	1 (0·3%)	8 (4·1%)	93 (4·1%)	3 (6·6%)	22 (3·1%)	4 (1·8%)	1 (0·6%)	8 (2·0%)
Laid off	1 (0·2%)	7 (0·7%)	14 (2·4%)	0 (0%)	14 (2·5%)	1 (0·8%)	6 (1·0%)	0 (0%)	1 (0·6%)	9 (0·4%)	0 (0%)	4 (0·5%)	5 (2·4%)	0 (0%)	0 (0%)
Not in workforce	35 (6·5%)	24 (2·4%)	4 (0·7%)	23 (4·9%)	82 (15%)	1 (0·5%)	60 (9·8%)	2 (1·0%)	3 (1·3%)	396 (18%)	0 (0%)	7 (1·0%)	3 (1·6%)	2 (1·2%)	0 (0%)
Retired	68 (13%)	60 (5·9%)	0 (0%)	32 (7·1%)	3 (0·6%)	0 (0%)	46 (7·5%)	22 (9·1%)	4 (1·9%)	87 (3·9%)	10 (22%)	97 (13%)	31 (15%)	15 (8·2%)	37 (9·3%)
Self-employed	53 (10·0%)	132 (13%)	28 (4·8%)	61 (13%)	56 (10%)	12 (8·7%)	11 (1·7%)	4 (1·6%)	41 (21%)	324 (14%)	8 (18%)	75 (10%)	8 (4·0%)	12 (6·6%)	20 (5·0%)
Student	99 (18%)	145 (14%)	134 (23%)	70 (15%)	131 (24%)	23 (17%)	81 (13%)	42 (18%)	32 (16%)	150 (6·7%)	4 (10%)	89 (12%)	32 (15%)	21 (11%)	49 (12%)
Working (>=40hrs/wk)	203 (38%)	409 (40%)	221 (38%)	202 (44%)	82 (15%)	64 (47%)	191 (31%)	142 (60%)	83 (43%)	891 (40%)	12 (27%)	307 (43%)	54 (25%)	87 (47%)	217 (54%)
Working (1-39hrs/wk)	60 (11%)	211 (21%)	129 (22%)	50 (11%)	137 (25%)	23 (17%)	165 (27%)	25 (11%)	22 (11%)	289 (13%)	8 (17%)	121 (17%)	74 (35%)	45 (25%)	69 (17%)
Height (m)	1·62 (0·08) (1·40 to 1·85)	1·66 (0·10) (1·23 to 2·10)	1·65 (0·17) (1·20 to 2·20)	1·61 (0·11) (1·15 to 1·96)	1·67 (0·12) (1·12 to 2·00)	1·66 (0·09) (1·40 to 1·93)	1·68 (0·09) (1·35 to 1·95)	1·67 (0·11) (1·48 to 1·85)	1·65 (0·13) (1·10 to 1·96)	1·66 (0·08) (1·40 to 1·90)	1·69 (0·07) (1·50 to 1·85)	1·65 (0·08) (1·43 to 1·85)	1·69 (0·10) (1·25 to 1·95)	1·68 (0·07) (1·45 to 1·80)	1·61 (0·07) (1·45 to 1·84)
Unknown	0	2	20	5	9	6	2	0	1	0	0	1	0	15	0
Weight(kg)	66 (12) (35 to 98)	72 (15) (40 to 120)	64 (12) (25 to 100)	67 (15) (21 to 112)	72 (16) (35 to 120)	67 (12) (32 to 97)	77 (18) (35 to 145)	68 (13) (39 to 103)	71 (19) (40 to 140)	65 (12) (32 to 137)	73 (13) (49 to 100)	65 (14) (37 to 108)	75 (20) (16 to 160)	76 (16) (40 to 134)	58 (9) (38 to 88)
Unknown	0	3	4	0	0	0	2	0	0	0	0	3	0	14	0
BMI	25·1 (4·3) (14·3 to 39·4)	26·2 (4·3) (13·8 to 39·4)	24·4 (6·6) (11·2 to 51·7)	25·8 (5·2) (7·5 to 47·4)	25·9 (5·1) (10·8 to 38·3)	24·6 (3·7) (12·9 to 32·9)	27·3 (5·3) (12·9 to 53·5)	24·5 (4·0) (15·2 to 36·9)	25·9 (6·7) (14·3 to 56·7)	23·3 (3·3) (13·3 to 45·7)	25·6 (3·9) (18·0 to 36·3)	23·6 (4·2) (14·1 to 38·9)	26·3 (7·0) (6·2 to 50·6)	27·1 (5·9) (16·9 to 50·2)	22·4 (2·4) (14·7 to 32·9)
Unknown	0	5	22	5	9	6	2	0	2	0	0	3	0	17	0
BMI classification															
Severely underweight	14 (2·7%)	9 (0·9%)	28 (5·1%)	7 (1·4%)	9 (1·7%)	2 (1·6%)	4 (0·6%)	0 (0·1%)	2 (1·1%)	9 (0·4%)	0 (0%)	12 (1·7%)	8 (3·8%)	0 (0%)	1 (0·3%)

Underweight	13 (2·4%)	16 (1·6%)	40 (7·1%)	19 (4·1%)	20 (3·8%)	0 (0·3%)	15 (2·5%)	7 (3·1%)	14 (7·3%)	101 (4·5%)	1 (1·5%)	43 (6·0%)	4 (1·8%)	1 (0·6%)	20 (4·9%)
Normal weight	159 (30%)	370 (37%)	289 (52%)	122 (27%)	211 (40%)	70 (54%)	91 (15%)	97 (41%)	78 (41%)	983 (44%)	22 (49%)	304 (42%)	89 (42%)	65 (38%)	212 (53%)
Overweight	92 (17%)	434 (43%)	117 (21%)	65 (14%)	153 (29%)	47 (36%)	93 (15%)	37 (16%)	55 (29%)	530 (24%)	15 (32%)	134 (19%)	63 (30%)	68 (41%)	120 (30%)
Obesity	257 (48%)	182 (18%)	84 (15%)	239 (53%)	136 (26%)	11 (8·2%)	404 (67%)	96 (40%)	41 (22%)	614 (27%)	7 (17%)	226 (31%)	47 (22%)	34 (20%)	48 (12%)
Unknown	0	5	22	5	9	6	2	0	2	0	0	3	0	17	0
Pregnant															
Not applicable	274 (51%)	479 (47%)	290 (50%)	225 (49%)	276 (51%)	66 (49%)	384 (63%)	104 (44%)	97 (50%)	1,117 (50%)	18 (41%)	344 (48%)	103 (49%)	72 (39%)	190 (48%)
No	250 (47%)	533 (52%)	261 (45%)	229 (50%)	253 (47%)	63 (46%)	213 (35%)	132 (56%)	95 (49%)	1,115 (50%)	26 (59%)	377 (52%)	109 (51%)	110 (60%)	205 (51%)
Yes	11 (2·1%)	4 (0·4%)	28 (4·9%)	4 (0·8%)	9 (1·7%)	7 (5·3%)	12 (2·0%)	1 (0·6%)	0 (<0·1%)	6 (0·3%)	0 (0%)	2 (0·3%)	0 (0%)	2 (0·8%)	5 (1·4%)
Gestational week	24 (10) (12 to 35)	24 (0) (24 to 24)	26 (5) (17 to 34)	0 (0) (Inf to -Inf)	6 (0) (6 to 8)	6 (2) (4 to 9)	14 (10) (5 to 30)	10 (0) (10 to 10)	0 (0) (Inf to -Inf)	9 (2) (5 to 10)	0 (0) (Inf to -Inf)	17 (13) (6 to 36)	0 (0) (Inf to -Inf)	29 (4) (25 to 34)	31 (7) (15 to 36)
Unknown	527	1,015	574	457	529	133	606	236	192	2,232	45	721	212	183	397
Regular medical follow-up before COVID-19															
No	339 (63%)	595 (59%)	409 (70%)	325 (71%)	407 (76%)	118 (86%)	412 (68%)	127 (54%)	131 (68%)	1,264 (56%)	24 (54%)	405 (56%)	104 (49%)	73 (40%)	69 (17%)
Yes	196 (37%)	421 (41%)	171 (30%)	132 (29%)	131 (24%)	19 (14%)	198 (32%)	110 (46%)	61 (32%)	974 (44%)	20 (46%)	318 (44%)	108 (51%)	109 (60%)	332 (83%)
Unknown	0	0	0	0	0	0	0	0	0	0	0	0	0	2	0
Practising healthcare professional															
No	344 (64%)	387 (38%)	265 (46%)	323 (71%)	378 (70%)	73 (53%)	433 (71%)	101 (42%)	127 (66%)	2,163 (97%)	40 (90%)	649 (90%)	140 (66%)	88 (47%)	175 (44%)
Yes	191 (36%)	629 (62%)	315 (54%)	134 (29%)	160 (30%)	64 (47%)	176 (29%)	136 (58%)	65 (34%)	75 (3·4%)	4 (10·0%)	74 (10%)	72 (34%)	97 (53%)	226 (56%)
Unknown	0	0	0	0	0	0	0	0	0	0	0	0	0	0	0
Number of children less than 18 years old															
No	320 (60%)	756 (74%)	358 (62%)	283 (62%)	350 (65%)	72 (53%)	254 (42%)	165 (70%)	138 (72%)	1,646 (74%)	30 (68%)	613 (85%)	153 (72%)	134 (72%)	146 (36%)
Yes	215 (40%)	260 (26%)	221 (38%)	174 (38%)	188 (35%)	64 (47%)	356 (58%)	72 (30%)	54 (28%)	592 (26%)	14 (32%)	110 (15%)	59 (28%)	51 (28%)	255 (64%)
Number of children aged above 18	0·92 (1·42) (0·00 to 8·00)	0·44 (0·98) (0·00 to	1·03 (1·71) (0·00 to	0·66 (1·01) (0·00 to 4·00)	0·96 (1·53) (0·00 to 6·00)	1·00 (1·31) (0·00 to	1·62 (1·74) (0·00 to	0·59 (0·98) (0·00 to	0·45 (0·82)	0·42 (0·76) (0·00 to 5·00)	0·55 (0·88) (0·00 to	0·21 (0·56) (0·00 to 4·00)	0·46 (0·88) (0·00 to 6·00)	0·44 (0·81) (0·00 to	1·08 (0·95) (0·00 to

		11·00)	10·00)			5·00)	7·00)	4·00)	(0·00 to 3·00)		3·00)			4·00)	4·00)
Unknown	0	14	5	3	10	0	17	0	0	0	0	2	2	0	0
Number of people lived with	4·63 (2·24) (1·00 to 16·00)	3·38 (1·76) (1·00 to 14·00)	4·74 (2·92) (1·00 to 23·00)	4·91 (2·30) (1·00 to 14·00)	6·28 (2·75) (1·00 to 18·00)	4·41 (2·22) (1·00 to 10·00)	6·00 (2·67) (1·00 to 21·00)	3·97 (1·61) (1·00 to 10·00)	3·96 (2·01) (1·00 to 12·00)	3·02 (1·13) (1·00 to 7·00)	2·98 (1·50) (1·00 to 6·00)	3·32 (1·83) (1·00 to 18·00)	2·67 (1·33) (1·00 to 8·00)	2·61 (1·26) (1·00 to 8·00)	4·18 (1·57) (1·00 to 12·00)
Unknown	1	4	5	5	49	0	28	0	3	0	0	1	0	16	1
Perceived social rank	3·55 (0·84) (1·00 to 5·00)	3·13 (0·66) (1·00 to 5·00)	3·20 (0·97) (1·00 to 5·00)	3·22 (0·71) (1·00 to 5·00)	3·16 (0·96) (1·00 to 5·00)	2·76 (0·80) (1·00 to 5·00)	3·45 (1·01) (1·00 to 5·00)	3·33 (0·73) (1·00 to 5·00)	3·46 (0·87) (1·00 to 5·00)	2·96 (0·70) (1·00 to 5·00)	3·33 (0·78) (1·00 to 5·00)	3·98 (0·83) (1·00 to 5·00)	3·43 (0·79) (1·00 to 5·00)	3·85 (0·83) (1·00 to 5·00)	3·21 (0·76) (1·00 to 5·00)
Unknown	0	0	0	0	0	0	0	0	0	0	0	0	0	11	0
House size	154 (136) (1 to 594)	137 (100) (10 to 500)	73 (77) (1 to 555)	121 (104) (1 to 561)	253 (145) (10 to 500)	104 (44) (12 to 249)	251 (157) (10 to 555)	102 (68) (1 to 500)	169 (122) (1 to 550)	32 (13) (10 to 100)	139 (71) (25 to 300)	151 (128) (10 to 540)	138 (119) (1 to 500)	196 (111) (20 to 560)	186 (143) (10 to 650)
Unknown	19	52	24	22	168	0	245	1	5	1	0	43	17	35	7

[1]n (%); Mean (SD) (Range)

eTable 4. Description of respondents' weighted fear of 11 factors by country.

Country	COVID-19		Cancer		Stroke		Heart Attack		Traffic accident		No social life		Crisis		Loss of family members		Animals/Insects		Loss of mobile phone		Job loss	
	Mean	SD	Mean	SD	Mean	SD	Mean	SD	Mean	SD	Mean	SD	Mean	SD	Mean	SD	Mean	SD	Mean	SD	Mean	SD
All sample	6.23	2.92	7.00	3.09	6.61	3.24	6.55	3.20	6.35	3.10	4.83	3.13	5.64	3.38	7.46	3.04	3.72	2.96	4.27	2.98	5.43	3.31
Australia	4.03	2.33	2.91	2.83	2.62	2.89	2.61	2.78	2.05	2.55	3.16	2.19	1.24	2.57	2.39	3.37	1.04	2.04	2.15	2.92	3.82	3.23
Brazil	7.22	2.67	6.86	3.46	5.83	3.70	5.77	3.61	5.71	3.39	4.44	3.17	3.81	3.58	7.76	2.77	3.00	3.03	3.13	2.90	4.75	3.73
Burundi	7.66	2.69	7.36	2.52	6.98	2.35	7.20	2.05	7.13	2.44	6.74	2.49	6.72	2.52	7.06	2.38	5.99	2.64	6.69	2.49	7.47	2.18
Canada	3.97	3.38	5.00	3.01	4.30	3.19	4.34	3.09	4.37	2.95	4.18	3.47	3.09	3.06	5.69	3.21	1.68	2.17	1.83	2.18	3.06	3.31
Chile	7.13	2.54	8.20	2.17	8.49	2.53	7.99	2.71	7.54	2.76	5.06	3.21	5.79	3.06	8.08	2.42	2.54	2.40	2.64	2.52	5.19	3.66
Egypt	3.32	2.84	4.20	3.23	3.99	3.28	4.02	3.19	3.95	3.08	3.03	2.92	3.32	3.13	4.79	3.31	2.73	2.98	2.79	2.98	2.90	2.91
Guatemala	7.09	2.46	8.21	2.64	7.91	2.83	7.36	2.96	6.67	2.71	3.09	2.85	5.73	3.10	8.20	2.50	3.41	2.77	2.98	2.55	6.22	3.47
Hong Kong	7.07	1.88	8.12	1.79	6.74	2.48	6.59	2.48	6.16	2.50	4.44	2.30	5.86	2.96	7.98	2.12	3.40	2.67	4.92	2.36	5.57	2.49
India	5.25	2.50	5.66	2.81	5.45	2.72	5.70	2.75	5.78	2.74	4.52	2.81	4.54	2.90	6.57	2.88	4.00	2.40	3.79	2.66	4.09	3.48
Indonesia	6.57	2.22	7.16	1.97	6.99	2.31	7.24	2.33	7.02	2.32	6.47	2.91	6.77	2.50	8.39	2.18	4.64	2.36	5.69	2.61	7.02	2.44
Italy	7.15	2.17	8.83	1.78	9.02	1.83	8.65	2.04	7.78	2.24	6.06	3.13	7.74	2.57	8.97	1.73	3.88	3.14	3.41	2.71	6.25	3.42
Lebanon	3.30	2.90	4.50	3.80	4.34	3.75	4.42	3.55	3.81	3.22	3.31	2.94	4.31	3.41	5.14	3.49	2.55	2.77	2.80	2.75	3.77	3.09
Libya	4.30	2.91	6.21	3.64	5.72	3.73	5.75	3.71	5.72	3.32	5.21	3.74	4.66	3.56	7.64	3.44	3.99	3.47	4.69	3.52	4.72	3.61
Macau	6.56	2.33	7.61	2.52	7.34	2.53	6.87	2.40	6.86	2.63	4.90	2.57	7.03	2.86	8.46	2.40	4.20	2.70	5.54	2.79	6.08	2.87
Mainland China	5.74	2.63	6.96	2.67	6.22	2.93	6.01	2.77	6.52	2.83	3.84	2.60	5.92	2.84	8.01	2.62	3.54	2.60	4.20	2.50	5.03	2.88
Malaysia	7.05	2.50	7.70	2.60	7.77	2.40	7.95	2.33	7.84	2.34	5.62	2.88	7.30	2.75	8.31	2.23	4.93	2.88	5.08	3.07	6.42	3.23
Mexico	7.37	2.53	7.86	2.77	7.81	2.98	7.56	2.97	6.85	2.92	3.43	3.20	6.12	3.25	8.04	2.59	3.43	2.97	3.16	2.85	6.26	3.37
Nigeria	4.94	3.03	5.98	3.37	5.65	3.32	5.90	3.43	6.10	3.22	4.40	3.05	5.98	3.38	6.89	3.20	4.32	2.99	4.76	3.07	5.72	3.35
Philippines	6.98	2.80	7.13	3.02	7.02	2.92	7.08	3.05	6.57	2.93	4.14	2.67	6.89	2.78	8.32	2.35	4.28	2.81	4.85	2.85	6.77	3.11
Republic Of Sudan	4.13	3.21	6.07	3.76	5.88	3.77	5.95	3.77	6.30	3.60	5.79	3.77	5.22	3.86	7.47	3.42	3.34	3.53	4.75	3.60	4.64	3.54
Rwanda	4.86	2.96	6.05	3.36	5.73	3.65	5.49	3.36	5.73	3.12	3.75	2.88	4.70	3.62	6.61	3.29	3.32	3.03	3.04	2.90	5.73	3.38
Saudi Arabia	4.45	2.91	6.49	3.62	6.35	3.68	6.37	3.67	6.22	3.37	5.32	3.62	5.23	3.79	7.16	3.44	3.88	3.33	4.29	3.14	5.33	3.57
Singapore	5.35	2.64	6.59	2.71	6.83	2.56	6.74	2.58	6.00	2.73	4.51	2.67	5.27	3.31	7.48	2.67	3.71	2.96	4.53	2.97	5.61	3.15
South Africa	6.09	3.01	7.48	2.67	6.78	3.02	7.08	2.99	6.76	2.83	3.02	2.87	6.15	3.31	8.12	2.61	3.98	2.65	4.29	3.10	6.84	3.16

South Korea	8.12	1.92	8.57	1.87	8.59	1.88	8.56	1.94	8.12	2.04	6.90	2.43	7.53	2.32	8.69	1.95	4.82	2.61	5.36	2.45	6.59	2.69
Spain	5.45	2.64	7.15	2.83	7.40	3.17	7.08	3.05	6.61	2.61	5.87	2.72	5.39	3.48	7.48	2.68	4.03	3.12	3.21	2.65	4.90	3.44
Thailand	6.41	2.70	6.41	3.14	6.00	3.26	5.83	3.18	6.62	2.82	3.51	2.89	4.61	3.23	6.87	3.20	4.11	2.80	4.54	2.99	4.53	3.75
United Kingdom	6.30	2.60	6.89	3.07	6.36	3.23	6.16	3.12	5.31	3.41	5.12	3.10	4.04	3.38	7.45	2.94	3.65	3.06	3.04	2.57	4.10	3.10
United States	6.06	2.73	7.24	2.69	6.42	3.20	6.17	3.10	6.31	2.81	4.27	3.01	5.64	3.09	7.35	2.68	3.28	2.78	4.79	2.99	3.29	2.86
Vietnam	6.76	2.70	7.93	2.51	7.71	2.68	7.43	2.71	7.76	2.71	6.87	2.92	7.24	2.87	8.42	2.58	3.58	2.96	3.61	2.80	5.96	3.17

eFigure 1. Visualization of the weighted fear of 11 factors by country, World Health Organization (WHO) region, economic development level, and COVID-19 severity level.

a. Weighted mean of fear of 11 factors by country.

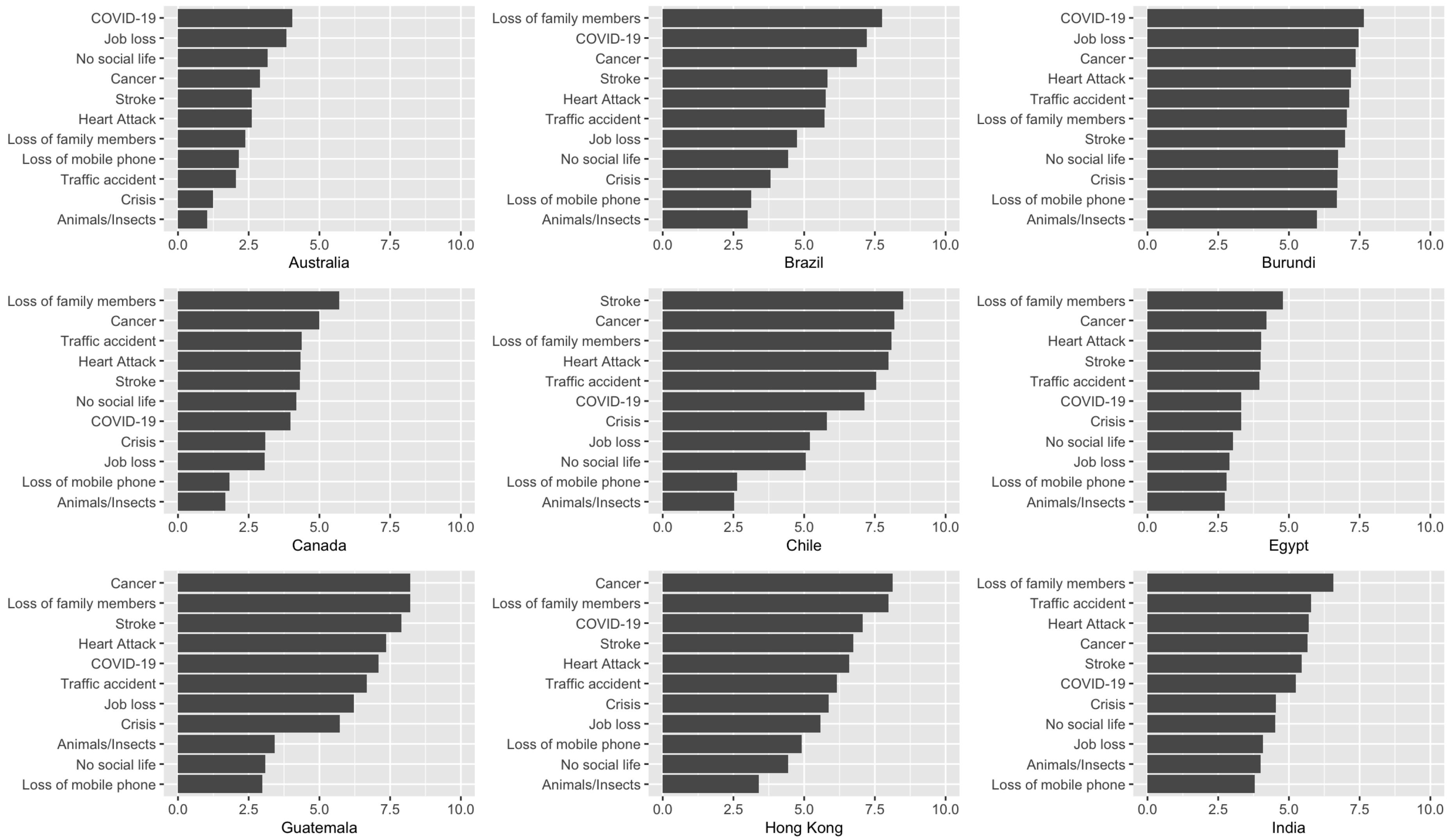

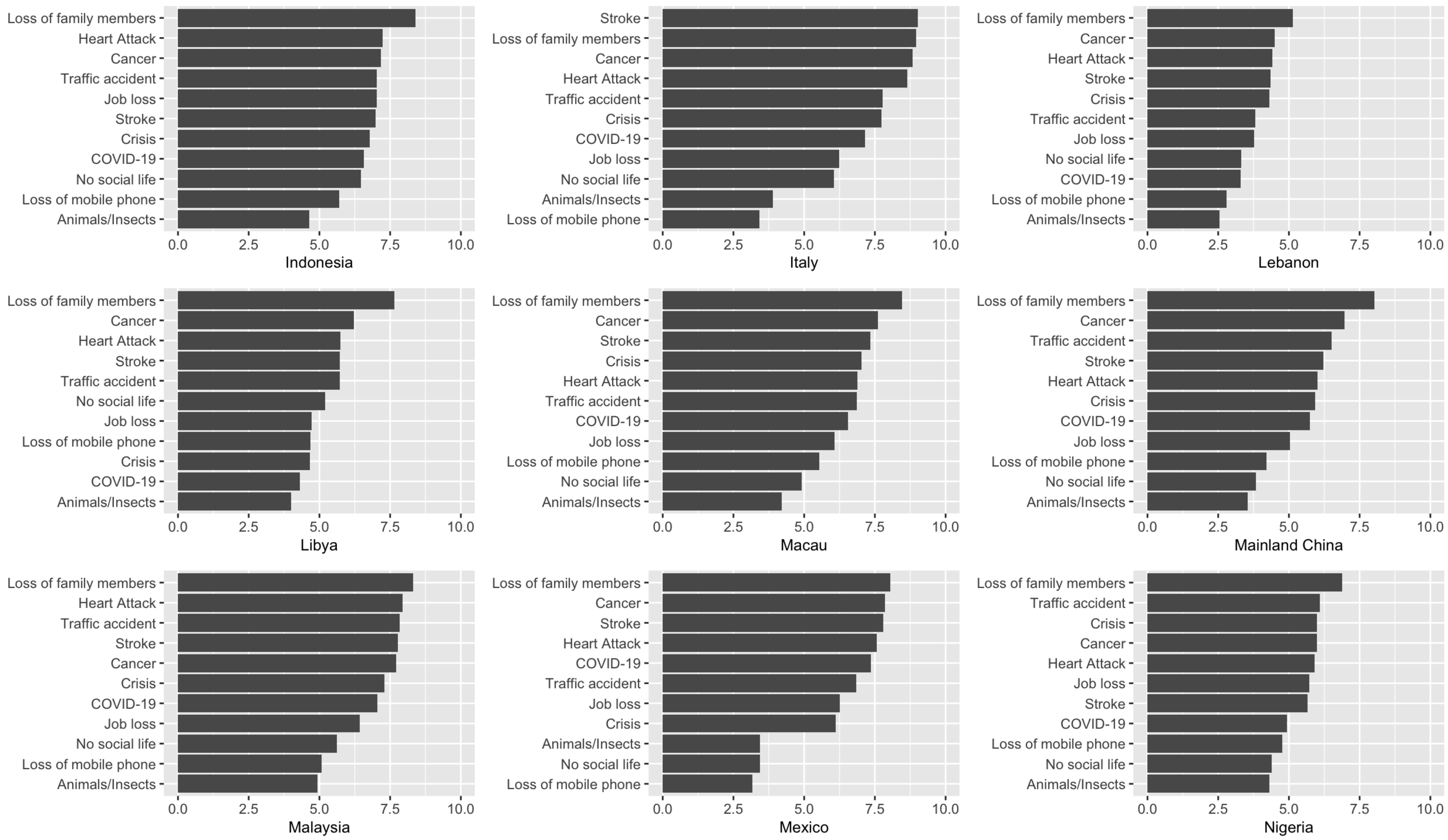
Loss of family members
Heart Attack
Cancer
Traffic accident
Job loss
Stroke
Crisis
COVID-19
No social life
Loss of mobile phone
Animals/Insects
0.0 2.5 5.0 7.5 10.0
Indonesia
Stroke
Loss of family members
Cancer
Heart Attack
Traffic accident
Crisis
COVID-19
Job loss
No social life
Animals/Insects
Loss of mobile phone
0.0 2.5 5.0 7.5 10.0
Italy
Loss of family members
Cancer
Heart Attack
Stroke
Crisis
Traffic accident
Job loss
No social life
COVID-19
Loss of mobile phone
Animals/Insects
0.0 2.5 5.0 7.5 10.0
Lebanon
Loss of family members
Cancer
Heart Attack
Stroke
Traffic accident
No social life
Job loss
Loss of mobile phone
Crisis
COVID-19
Animals/Insects
0.0 2.5 5.0 7.5 10.0
Libya
Loss of family members
Cancer
Stroke
Crisis
Heart Attack
Traffic accident
COVID-19
Job loss
Loss of mobile phone
No social life
Animals/Insects
0.0 2.5 5.0 7.5 10.0
Macau
Loss of family members
Cancer
Traffic accident
Stroke
Heart Attack
Crisis
COVID-19
Job loss
Loss of mobile phone
No social life
Animals/Insects
0.0 2.5 5.0 7.5 10.0
Mainland China
Loss of family members
Heart Attack
Traffic accident
Stroke
Cancer
Crisis
COVID-19
Job loss
No social life
Loss of mobile phone
Animals/Insects
0.0 2.5 5.0 7.5 10.0
Malaysia
Loss of family members
Cancer
Stroke
Heart Attack
COVID-19
Traffic accident
Job loss
Crisis
Animals/Insects
No social life
Loss of mobile phone
0.0 2.5 5.0 7.5 10.0
Mexico
Loss of family members
Traffic accident
Crisis
Cancer
Heart Attack
Job loss
Stroke
COVID-19
Loss of mobile phone
No social life
Animals/Insects
0.0 2.5 5.0 7.5 10.0
Nigeria

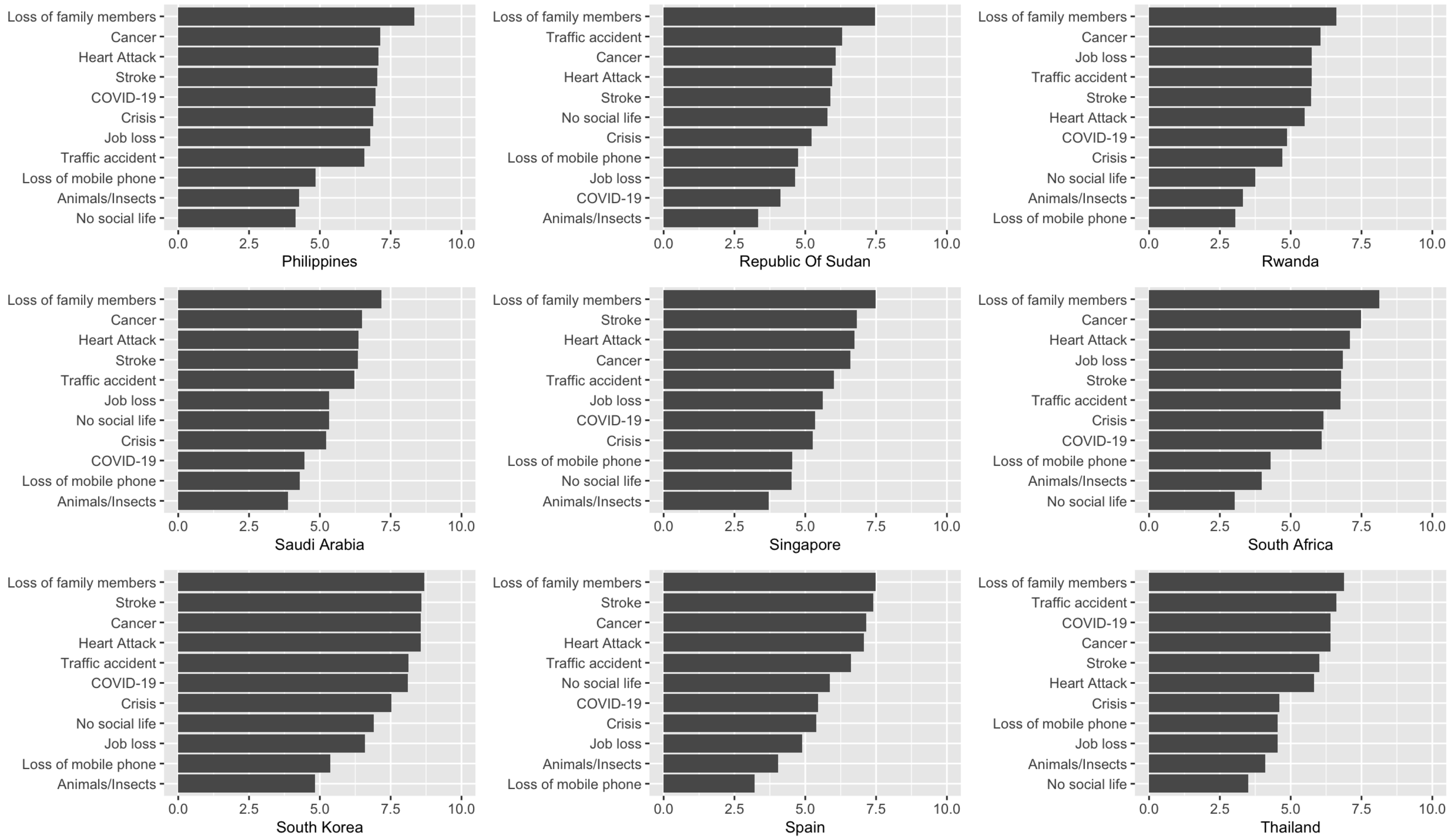

Loss of family members
Cancer
Heart Attack
Stroke
COVID-19
Crisis
Job loss
Traffic accident
Loss of mobile phone
Animals/Insects
No social life
0.0 2.5 5.0 7.5 10.0
Philippines
Loss of family members
Traffic accident
Cancer
Heart Attack
Stroke
No social life
Crisis
Loss of mobile phone
Job loss
COVID-19
Animals/Insects
0.0 2.5 5.0 7.5 10.0
Republic Of Sudan
Loss of family members
Cancer
Job loss
Traffic accident
Stroke
Heart Attack
COVID-19
Crisis
No social life
Animals/Insects
Loss of mobile phone
0.0 2.5 5.0 7.5 10.0
Rwanda
Loss of family members
Cancer
Heart Attack
Stroke
Traffic accident
Job loss
No social life
Crisis
COVID-19
Loss of mobile phone
Animals/Insects
0.0 2.5 5.0 7.5 10.0
Saudi Arabia
Loss of family members
Stroke
Heart Attack
Cancer
Traffic accident
Job loss
COVID-19
Crisis
Loss of mobile phone
No social life
Animals/Insects
0.0 2.5 5.0 7.5 10.0
Singapore
Loss of family members
Cancer
Heart Attack
Job loss
Stroke
Traffic accident
Crisis
COVID-19
Loss of mobile phone
Animals/Insects
No social life
0.0 2.5 5.0 7.5 10.0
South Africa
Loss of family members
Stroke
Cancer
Heart Attack
Traffic accident
COVID-19
Crisis
No social life
Job loss
Loss of mobile phone
Animals/Insects
0.0 2.5 5.0 7.5 10.0
South Korea
Loss of family members
Stroke
Cancer
Heart Attack
Traffic accident
No social life
COVID-19
Crisis
Job loss
Animals/Insects
Loss of mobile phone
0.0 2.5 5.0 7.5 10.0
Spain
Loss of family members
Traffic accident
COVID-19
Cancer
Stroke
Heart Attack
Crisis
Loss of mobile phone
Job loss
Animals/Insects
No social life
0.0 2.5 5.0 7.5 10.0
Thailand

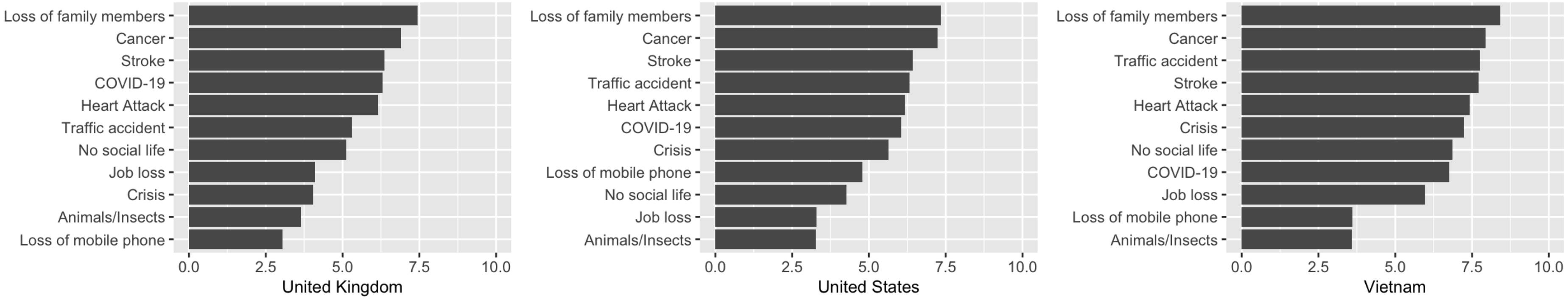

Loss of family members
Cancer
Stroke
COVID-19
Heart Attack
Traffic accident
No social life
Job loss
Crisis
Animals/Insects
Loss of mobile phone
0.0
2.5
5.0
7.5
10.0
United Kingdom
Loss of family members
Cancer
Stroke
Traffic accident
Heart Attack
COVID-19
Crisis
Loss of mobile phone
No social life
Job loss
Animals/Insects
United States
Loss of family members
Cancer
Traffic accident
Stroke
Heart Attack
Crisis
No social life
COVID-19
Job loss
Loss of mobile phone
Animals/Insects
Vietnam

b. Comparison of respondents' weighted mean of fear of 11 factors across countries.

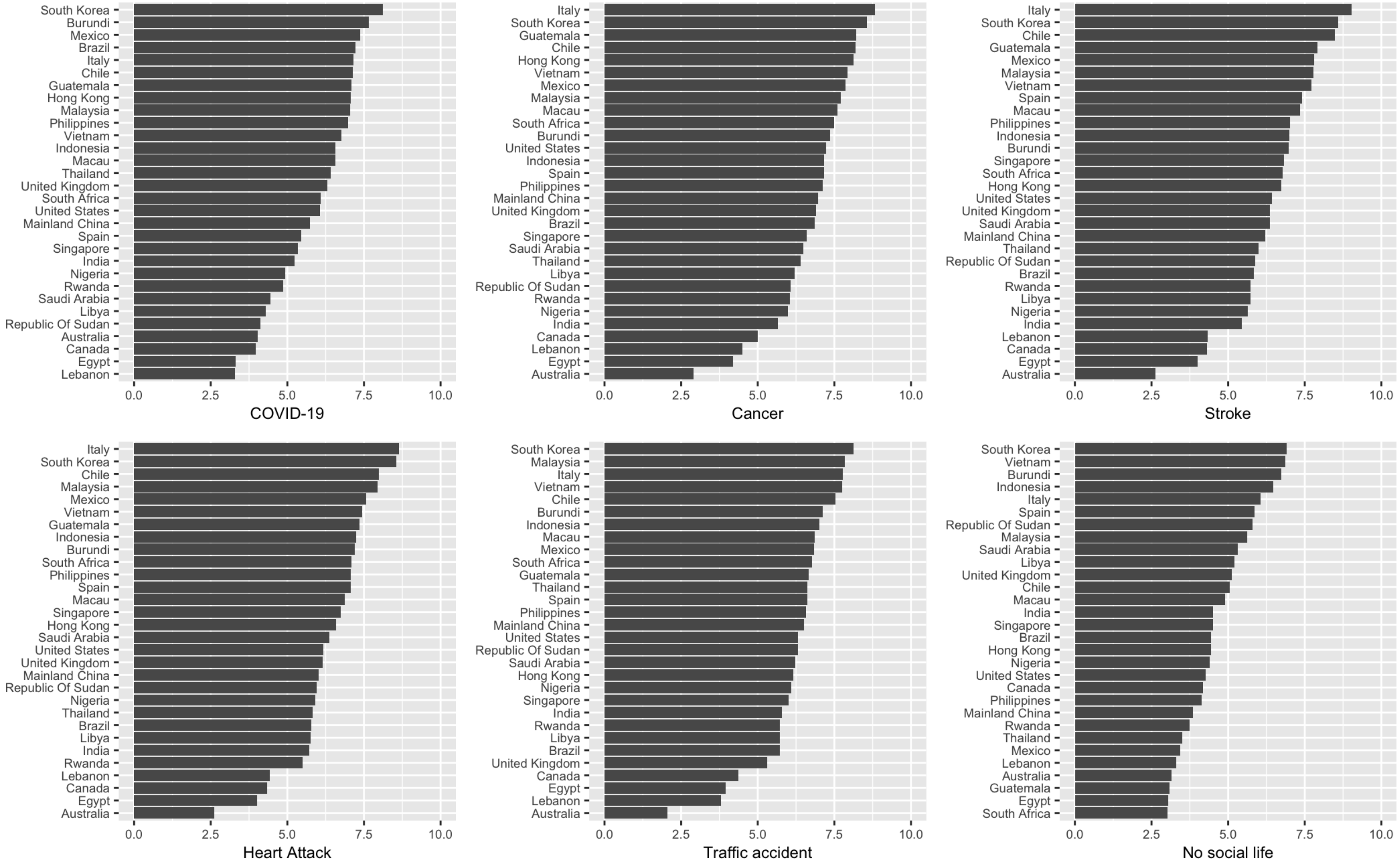

The picture can't be displayed.

c. Weighted mean of fear of 11 factors by region.

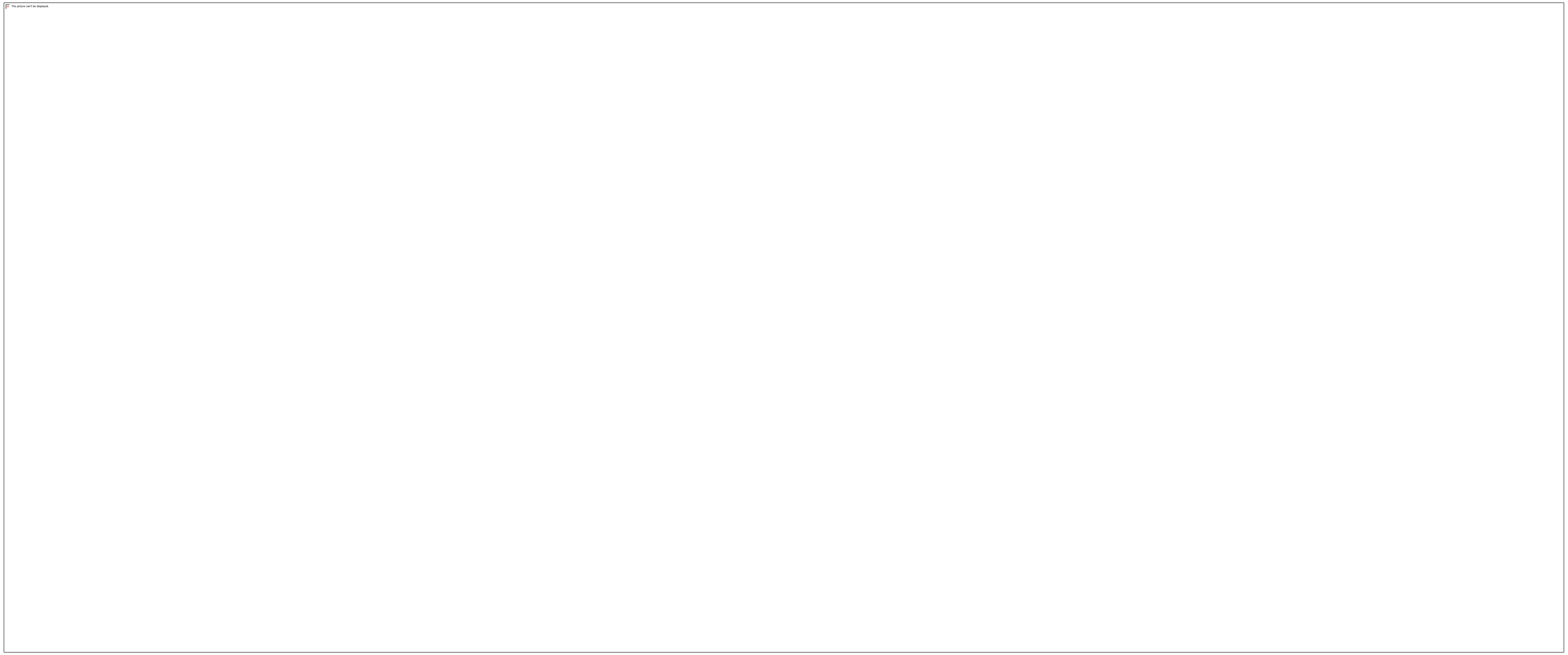

d. Weighted mean of fear of 11 factors by economic development level.

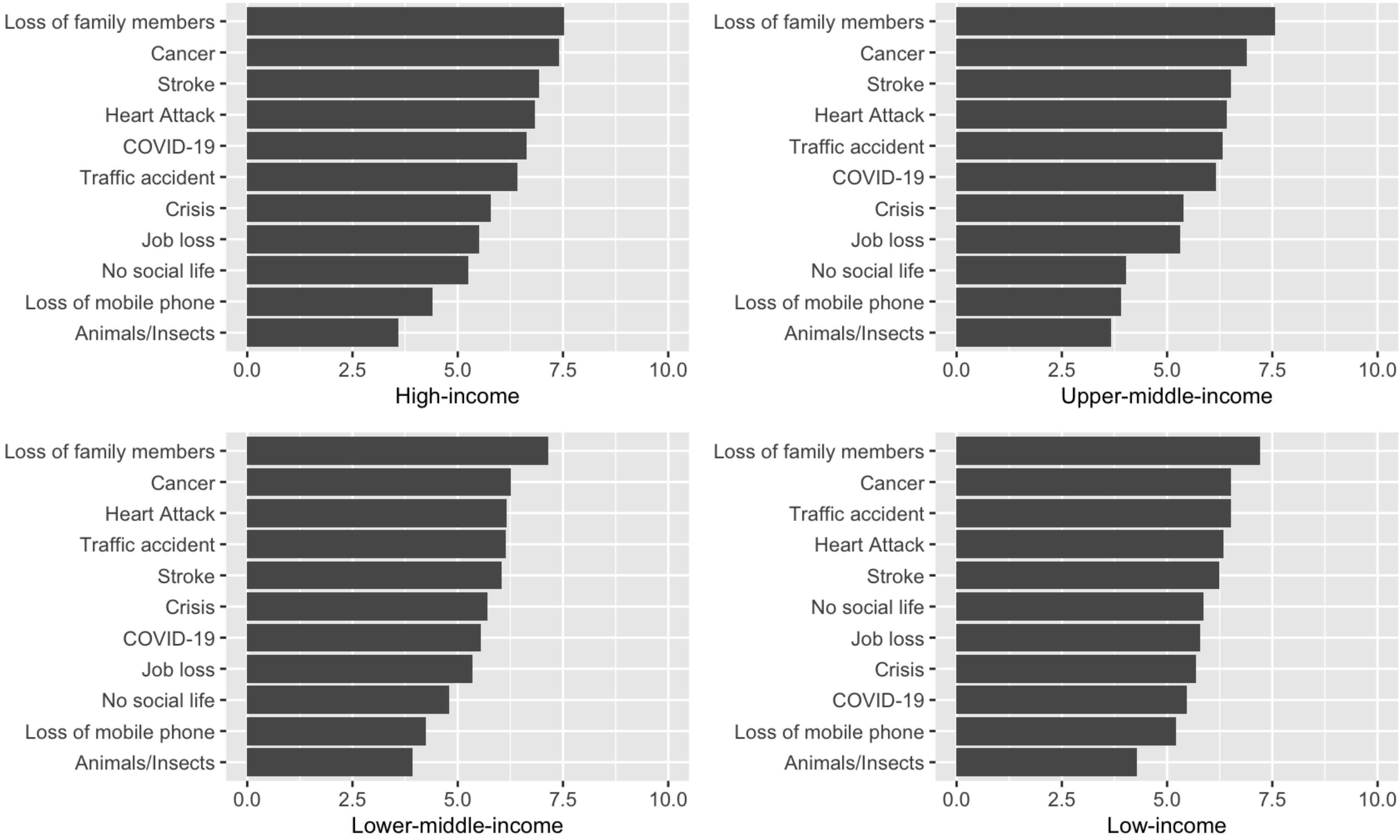

e. Weighted mean of fear of factors by COVID-19 severity level.

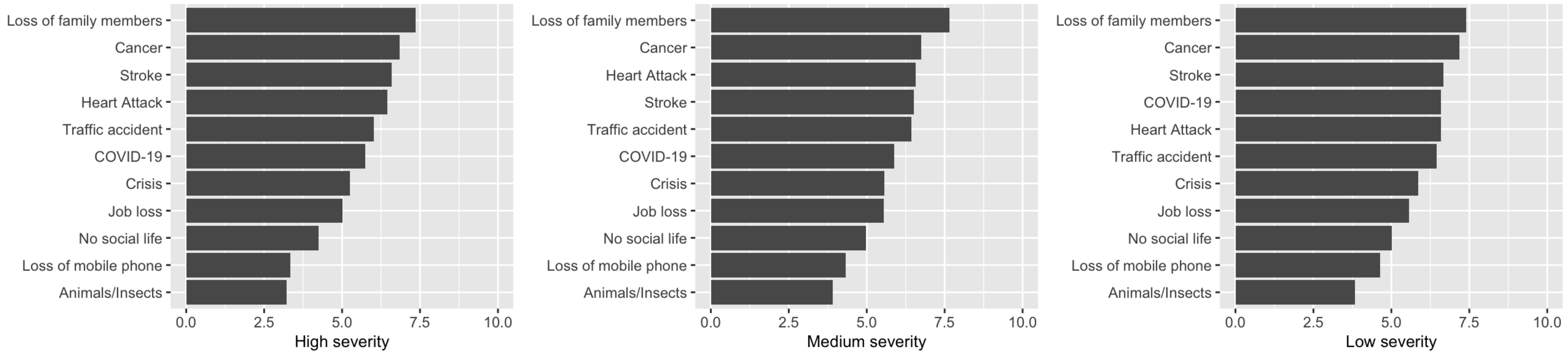

Supplement: Online Supplementary Document. [file jogh-14-05019-s001.pdf]
